# Association of KIR2DL5, KIR2DS5, and KIR2DS1 allelic variation and atopic dermatitis

**DOI:** 10.1038/s41598-023-28847-y

**Published:** 2023-01-31

**Authors:** David J. Margolis, Nandita Mitra, Ole J. Hoffstad, Ronald Berna, Brian S. Kim, Abha Chopra, Elizabeth J. Phillips

**Affiliations:** 1 Department of Biostatistics, Epidemiology and Informatics, Perelman School of Medicine, Philadelphia, PA USA; 2 Department of Dermatology, Perelman School of Medicine, University of Pennsylvania, 901 Blockley Hall, 423 Guardian Drive, Philadelphia, PA 19104 USA; 3 Department of Dermatology, Icahn School of Medicine at Mount Sinai, New York, NY USA; 4 Institute for Immunology and Infectious Diseases, Murdoch University, Murdoch, Australia; 5 Center for Drug Safety and Immunology, Vanderbilt University Medical Center, Nashville, TN USA

**Keywords:** Skin diseases, Disease genetics, Diagnostic markers

## Abstract

Natural killer cells (NK) have been associated with the pathophysiology of atopic dermatitis (AD). NK function is regulated by killer cell Ig-like receptor family (KIR) receptors that interact with HLA ligands. The study goal was to focus on allelic variation in genes *KIR2DL5*, *KIR2DS5*, and *KIR2DS1* with respect to AD. This was a case–control study of individuals with (n = 313) and without (n = 176) AD. Associations were estimated using logistic regression. The prevalence of *KIR2DL5* was 52.5% (95% CI 48.0,57.0), *KIR2DS5* was 33.0% (28.8,37.3), and *KIR2DS1* was 33.6% (29.4,38.0). The presence of the KIR2DL5*001:01 increased the odds of having AD by about 86% (odds ratio (OR): 1.86(1.23,2.82) p = 0.003). The risk for individuals homozygous for KIR2DL5*001:01 was even greater (OR: 2.16 (95% CI 1.31,3.53) p = 0.0023). The odds of having AD with KIR2DL5*001:01 was similar in Whites and Blacks. Allelic variation in *KIR2DS5* and *KIR2DS1* was not associated with AD. There is no known HLA binding ligand for *KIR2DL5*. The effect of KIR2DL5*001:01 increased in the presence of HLA-B*-21TT leader sequence (2.46(1.37,4.41) p = 0.0025) and the HLA-C2 ligand (2.07 (1.37,4.41, p = 0.000002). Our study shows an independent association of the KIR2DL5*001:01 with AD and is the first study to associate AD with KIR allelic variation.

## Introduction

Atopic dermatitis (AD) is a common, pruritic, inflammatory skin disease characterized by life-long periods of acute disease flares as well as remissions that has been associated with genetic variation^1–6^. AD is associated with T-cell dysregulation^7–10^. Several recent studies, have associated AD with altered numbers of natural killer (NK) cells and diminished NK cell function^11–16^. NK cell function is directly associated with Human Leukocyte Antigen (HLA) Class I and NK cell surface receptors like the killer cell Ig-like receptor family (KIR) as well as other NK cell surface receptors^17–22^. NK cells are lymphocytes that have classically been described as part of the innate immune system but NK cells also have adaptive immune properties^18,23–25^. NK cell receptors recognize highly conserved nonpolymorphic HLA class I leader sequence sites as well as polymorphic HLA Class I ligands^24–27^. NK cell functions include the identification and removal of virally infected cells and malignant clones^12,25,27^.

The NK cell membrane bound KIR ligands are the primary regulator of NK cell function^18,20,21^. There are up to fifteen *KIR* genes found on chromosome 19 that code for the KIR ligands that are simply thought to have either activating (usually denoted by “S” for short tail, e.g., *KIR2DS1*) or inhibitory functions (usually denoted by “L” for long tail, e.g., *KIR2DL5*) for NK cells^18,26,28,29^. Most mature NK cells express KIR, although the total number of *KIR* genes observed and expressed as well as the type of KIR ligands produced by the genes (i.e., inhibitory and activating ligands) varies by individual^18,20,21^. We have recently shown that the *KIR* genes *KIR2DL5*, *KIR2DS5*, and *KIR2DS1* are more frequently found in individuals with AD^22^. This is also true for the HLA-C2 epitope and HLA-B*-21T leader sequence^22^.

The goal of this study was to examine allelic variation in *KIR* genes *KIR2DL5*, *KIR2DS5*, and *KIR2DS1* that were previously associated with AD^22,30,31^. This investigation is important in that the pathophysiology of AD has not been fully elucidated and dysregulation of immune function is being actively investigated in the quest for new therapeutics.

## Results

### Study cohort

We focused on the allelic variation of the three *KIR* genes previously shown to be associated with AD; *KIR2DL5*, *KIR2DS5*, and *KIR2DS1*^22^. Sufficient DNA for KIR typing was available from 506 Genetic of Atopic Dermatology (GAD) cohort participants. The KIR typing was successful for 489 (96.6%) subjects. Frequencies for all KIR gene alleles are provided in the supplement. Among those with full allelic typing, there were 313 (64.1%) AD cases and 176 (35.9%) controls. The AD cases included 167 (53.0%) Whites and 125 (40.0%) Blacks. The controls included 114 (65.1%) Whites and 59 (33.7%) Blacks. Of those with AD, 175 (56.6%) had asthma and 194 (62.8%) had seasonal allergies (4 individuals were missing this information). The median age of onset of AD was 0.75 years (IQR: 0.25–7.0). On the day of enrollment, about 25% of the subjects in the GAD case group had moderate to severe AD based on the patient-oriented eczema measure (POEM), with a group mean POEM score of 7.31 (6.65, 7.90)^32^.

### KIR gene prevalence

The overall prevalence of *KIR2DL5* gene was 52.5% (95% CI 48.0,57.0), *KIR2DS5* gene was 33.0% (28.8,37.3), and *KIR2DS1* gene was 33.6% (29.4,38.0) in the GAD cohort. As previously reported, subjects with these genes were more likely to have AD; *KIR2DL5* (OR:1.57 95% CI (1.07,2.31)), *KIR2DS5* (1.80 (1.18,2.76)), and *KIR2DS1* (1.57 (1.03,2.39)). The copy number variation (CNV) of *KIR2DL5* and *KIR2DS5* varied from 0 to 4 copies and increasing CNV was associated with AD (1.52(1.22,1.89), *p* < 0.0001 and 1.66(1.16,2.37), *p* = 0.006, respectively). The *KIR2DL5* was not highly correlated (*R*^2^ approximately 0.45) to the two other KIR genes. *KIR2DS1* and *KIR2DS5* were moderately in linkage disequilibrium (LD) (*R*^2^ approximately 0.61). The total number of alleles discovered by genotyping for *KIR2DL5* was 10, *KIR2DS5* was 9, and *KIR2DS1* was 8 (see Supplement for all KIR gene frequencies). As noted in the methods section, a priori we decided to evaluate only those alleles with an allelic frequency of ≥ 0.05 (Table 1). The number of alleles meeting this criterion for *KIR2DL5* was 2, *KIR2DS5* was 1, and *KIR2DS1* was 3 (Table 1).

**Table 1:** Allelic frequencies ≥ 0.05 for 2DL5, 2DS1, and 2DS5.

Allele	Full cohort		White only		Black only	
	n	AF (95% CI)	n	AF (95% CI)	n	AF (95% CI)
KIR2DL5*001:01	277	0.28 (0.25,0.31)	145	0.26 (0.22,0.30)	115	0.31 (0.27,0.36)
KIR2DL5*002:01	224	0.23 (0.20,0.26)	137	0.24 (0.21,0.28)	71	0.19 (0.15,0.24)
KIR2DS1*002:01	308	0.31 (0.28,0.34)	209	0.37 (0.33,0.41)	73	0.20 (0.16,0.24)
KIR2DS5*002:01	244	0.25 (0.22,0.28)	172	0.31 (0.27,0.35)	54	0.15 (0.11,0.19)
KIR2DS5*005:01	31	0.03 (0.02,0.04)	2	0.00 (0.00,0.01)	29	0.08 (0.05,0.11)
KIR2DS5*009	19	0.02 (0.01,0.03)	2	0.00 (0.00,0.01)	17	0.05 (0.03,0.07)
Atopic dermatitis (cases)						
KIR2DL5*001:01	204	0.33 (0.29,0.36)	97	0.29 (0.24,0.34)	92	0.37 (0.31,0.43)
KIR2DL5*002:01	141	0.23 (0.19,0.26)	79	0.24 (0.19,0.29)	48	0.19 (0.15,0.25)
KIR2DS1*002:01	207	0.33 (0.29,0.37)	135	0.41 (0.35,0.46)	50	0.20 (0.15,0.26)
KIR2DS5*002:01	165	0.26 (0.23,0.30)	114	0.34 (0.29,0.40)	37	0.15 (0.11,0.20)
KIR2DS5*005:01	31	0.05 (0.03,0.07)	2	0.01 (0.00,0.02)	29	0.12 (0.08,0.16)
KIR2DS5*009	15	0.02 (0.01,0.04)	0	0.00 (0.00,0.01)	15	0.06 (0.03,0.10)
Controls						
KIR2DL5*001:01	72	0.20 (0.16,0.25)	48	0.21 (0.16,0.27)	23	0.19 (0.13,0.28)
KIR2DL5*002:01	82	0.23 (0.19,0.28)	58	0.25 (0.20,0.31)	23	0.19 (0.13,0.28)
KIR2DS1*002:01	99	0.28 (0.23,0.33)	74	0.32 (0.26,0.39)	23	0.19 (0.13,0.28)
KIR2DS5*002:01	77	0.22 (0.18,0.27)	58	0.25 (0.20,0.31)	17	0.14 (0.09,0.22)
KIR2DS5*005:01	0	0.00 (0.00,0.01)	0	0.00 (0.00,0.02)	0	0.00 (0.00,0.03)
KIR2DS5*009	4	0.01 (0.00,0.03)	2	0.01 (0.00,0.03)	2	0.02 (0.00,0.06)

Allelic frequencies (AF) are presented with 95% CI for the full cohort or by race. n = sample size.

### KIR allele frequency

In the GAD cohort, the presence of the KIR2DL5*001:01 allele increased the odds of AD by about 86% (1.86(1.23,2.82) p = 0.003). More specifically, those homozygous for KIR2DL5*001:01 had an OR of 2.16 (1.31,3.53; p = 0.002) (Tables 2 and 3). The risk of having AD for KIR2DL5*001:01 was similar in Whites or Blacks (Table 3 and Supplementary Table 2). The alleles from the other *KIR* genes of interest were not associated with AD. KIR2DL5*001:01 is part of the *KIR2DL5A* gene complex which is located in the telomeric half of the *KIR* gene locus on chromosome 19 and can include the *KIR2DS1* and *KIR2DS5*^33,34^. Those with *KIR2DL5A* alleles were at an increased risk for having AD (1.86 (1.23, 2.82) p = 0.003) (Table 2).

**Table 2:** Odds ratios and 95% CI for the association of atopic dermatitis by KIR alleles of interest (allelic frequency of > 0.05).

Allele	Presence or absence of allele	Heterozygote	Homozygote
	OR (95% CI)	OR (95% CI)	OR (95% CI)
Full cohort			
KIR2DL5A	1.86 (1.23,2.82)^^^	1.47 (0.74,2.94)	2.16 (1.32,3.54)^^^
KIR2DL5B	0.99 (0.66,1.50)	1.47 (0.74,2.94)	1.16 (0.74,1.90)
KIR2DL5*002:01	0.95 (0.63,1.44)	1.07 (0.54,2.13)	0.90 (0.56,1.46)
KIR2DL5*001:01	1.86(1.23,2.82)^^^	1.54 (0.78,3.06)	2.16 (1.31,3.53)^^^
KIR2DS1*002:01	n/a	0.63 (0.04,10.21)	1.33 (0.89,2.00)
KIR2DS5*002:01	2.91 (1.24,6.80)*	1.97 (0.20,19.14)	1.40 (0.90,2.18)
KIR2DS5*005:01	1.41 (0.93,2.14)	n/a	n/a
White cohort			
KIR2DL5A	1.66 (0.98,2.82)	1.63 (0.73,3.68)	1.68 (0.91,3.12)
KIR2DL5B	0.98 (0.58,1.66)	1.39 (0.62,3.14)	0.81 (0.44,1.51)
KIR2DL5*002:01	0.98 (0.58,1.66)	1.44 (0.66,3.28)	0.78(0.42,1.46)
KIR2DL5*001:01	1.66 (0.98,2.82)	1.69 (0.74,3.85)	1.61 (0.85,3.07)
KIR2DS1*002:01	n/a	n/a	1.47 (0.89,2.43)
KIR2DS5*002:01	0.79 (0.05,12.77)	1.55 (0.91,2.63)	n/a
KIR2DS5*005:01	1.50 (0.88,2.53)	n/a	n/a
Black cohort			
KIR2DL5A	2.80 (1.24,6.32)*	1.63(0.41,7.18)	2.58 (1.18,5.63)*
KIR2DL5B	1.01 (0.50,2.05)	1.26 (0.32,4.99)	0.94 (0.43,2.06)
KIR2DL5*002:01	0.92 (0.44,1.97)	0.62 (0.13,2.88)	1.02 (0.45,2.33)
KIR2DL5*001:01	2.67 (1.24,5.74)*	1.78 (0.44,7.18)	2.80 (1.24,6.32)*
KIR2DS1*002:01	n/a	n/a	1.16 (0.53,2.56)
KIR2DS5*002:01	2.93 (1.13,7.60)*	1.76 (0.18,17.44)	1.25 (0.50,3.13)
KIR2DS5*005:01	1.37 (0.67,2.78)	n/a	n/a

Presence or absence of the allele of interest as well as the allele of interest as a heterozygote or homozygous (gene present and homozygote for the allele). For 2DL5 composite was presented for alleles near telomer (A) or centromere (B); A—KIR2DL5*001:01:01, KIR2DL5*001:02, KIR2DL5*001:03, KIR2DL5*001:09:01; B—KIR2DL5*002:01:01 KIR2DL5*002:03, KIR2DL5*003, KIR2DL5*004, KIR2DL5*013:01. n/a—unstable estimate due to insufficient sample size; *p < 0.05, ^^^p < 0.005.

**Table 3:** Odds ratios and 95% CI for the association of atopic dermatitis for KIR alleles of interest evaluated by the presence or absence of the allele.

Allele	Full cohort	Whites	Blacks
	OR (95% CI)	OR (95% CI)	OR (95% CI)
KIR2DL5A	1.85 (1.22,2.79)^^^	1.64 (0.96,2.77)	2.36 (1.16,4.81)*
KIR2DL5B	1.02 (0.68,1.53)	1.03 (0.61,1.73)	1.01 (0.50,2.05)
KIR2DL5*002:01	0.98 (0.65,1.48)	1.03 (0.61,1.73)	0.93 (0.44,1.97)
KIR2DL5*001:01	1.85 (1.22,2.79)^^^	1.64 (0.96,2.77)	2.36 (1.16,4.81)*
KIR2DS1*002:01	1.25 (0.84,1.88)	1.46 (0.89,2.41)	0.98 (0.45,2.12)
KIR2DS5*002:01	1.29 (0.83,1.99)	1.55 (0.91,2.63)	1.06 (0.45,2.49)
KIR2DS5*005:01	n/a	n/a	n/a

Only alleles with variant frequency ≥ 0.05. n/a—unstable estimate due to insufficient sample size. *p < 0.05, ^^^p < 0.005.

### KIR allele and HLA interactions

Interactions between HLA ligands and *KIR* alleles are presented for the presence or absence of the *KIR* allele and HLA ligand (Table 4). Similar results were obtained for the *KIR* alleles (categorized as heterozygote or homozygote). There is no known HLA ligand for *KIR2DL5.* A previous report showed an increased effect of *KIR2DL5* in the presence of HLA-B *-21TT^22^. The effect of KIR2DL5*001:01 increased in the presence of HLA-B *-21TT leader sequence (2.46 (1.37,4.41) p = 0.0025), C2 (2.07 (1.37,4.41)), and the weakly binding Bw4 epitope variant B80T (2.91(1.50,5.63) p = 0.0016). The association between AD and KIR2DL5*001:01 was not found to be confounded by race, HLA-A*01:01, HLA-A*02:01, HLA-B*07:02, HLA-C*07:02, C2, Bw4 (an HLA ligand), B80T (an HLA variant associated with HLA-B position 80 coding for threonine), seasonal allergies or asthma.

**Table 4:** ORs for the KIR allele with 95% CI by the presence or absence of known HLA KIR ligands.

HLA ligand	KIR2DL5*001:01		KIR2DS5*002:01		KIR2DS1*002:01		KIR2DL5*002:01	
	Present	Absent	Present	Absent	Present	Absent	Present	Absent
	OR (95% CI)	OR (95% CI)	OR (95% CI)	OR (95% CI)	OR (95% CI)	OR (95% CI)	OR (95% CI)	OR (95% CI)
B21TT	2.46 (1.37,4.41)^^^	1.34 (0.74,2.41)	1.64 (0.89,3.02)	0.97 (0.52,1.81)	1.68 (0.94,2.98)	0.92 (0.52,1.63)	1.24 (0.68,2.29)	0.79 (0.45,1.40)
C2	2.07 (1.25,3.42)^^^	1.48 (0.72,3.05)	1.30 (0.76,2.22)	1.26 (0.59,2.66)	1.44 (0.88,2.35)	0.95 (0.46,1.94)	1.51 (0.89,2.56)	0.44 (0.22,0.88)
C1	1.67 (1.04,2.68)*	2.53 (1.07,5.97)*	1.25 (0.76,2.05)	1.44 (0.57,3.62)	1.15 (0.73,1.81)	1.71 (0.72,4.10)	0.85 (0.53,1.36)	1.69 (0.65,4.40)

The HLA ligand associated with KIR2DL5 is currently unknown. KIR2DS1 recognizes HLA-C2 and specific KIR2DS5 allotypes have been previously shown to bind HLA-C2. HLA-B *-21TT has been previously associated with KIR binding. KIR2DS5*005:01 not estimated due to inadequate sample size. *p < 0.05, ^^^p < 0.005.

## Discussion

This is the first case–control study of AD to explore *KIR* **allelic** variation. As previously reported, the presence of three KIR genes is associated with AD; *KIR2DL5*, *KIR2DS1*, and *KIR2DS5*^22^. However, only the KIR2DL5*001:01 allele is independently associated with an increased risk of AD. This association is greatest for the presence of KIR2DL5*001:01. The other *KIR2DL5* variants did not increase the risk of AD. This is consistent with recent studies that have demonstrated that the KIR2DL5*002:01, which is the second most frequent and is the dominant centromeric *KIR2DL5* variant, is silenced and not expressed on the NK cell surface^35,36^. The increased risk associated with KIR2DL5*001:01 is augmented by an interaction with HLA-B*-21TT leader sequence, which is associated with less “educated” or less active NK cells^22,24^. The magnitude of the effect of KIR2DL5*001:01 may also be augmented by HLA-C epitope C2.

Our findings are consistent with other diseases. Previous studies of HIV have shown that within a *KIR* gene, *KIR* allelic variation can have important influences on the functioning of NK cells as well as how the KIR receptor interacts with HLA ligand^37^. In addition our findings refine our previous investigation, which was based on simply genotyping the presence or absence of the *KIR* gene rather than focusing on specific alleles^22^. The risk of AD due to KIR2DL5*001 likely does not vary significantly by race; although the effect of KIR2DL5*001:01 may be greater in Blacks with AD than Whites.

Two recent studies described the potential physiology of an association between AD and NK cell function and number^11,16^. In a cohort of individuals with moderate to severe AD by *Mack *et al. , individuals with AD had fewer circulating NK cells that are more homogenous than expected^11^. The number of circulating NK cells could be returned to control subject levels after treatment with anti-IL-4 blockade and IL-15 super-agonism^11^. Furthermore, Mack et al. showed a low frequency of *KIR2DL5* cell markers on the circulating NK cells but did not compare the frequency of these cell markers between groups with and without AD. Some of the subjects evaluated by Mack et al. were from the GAD cohort. Mobus et al. evaluated NK cells using skin transcriptomics in a likely White European group with moderate to severe AD^16^. These investigations noted an increased number of NK cells in AD lesional skin as compared to AD non-lesional skin or healthy control skin^16^. Mobus et al. further showed that surface markers on the NK cells from AD lesional skin consistently had an increase in inhibitory receptors and were poorly educated^16^. There findings are consistent with KIR2DL5*001:01 function. Their findings could be reversed after treatment with anti-IL-4 blockade and calcineurin inhibition^16^. However, the inhibitory receptors evaluated by these investigators were not *KIR* receptors but natural cytotoxicity receptors (NCR)^16,38,39^. While Mobus et al. implied that their results contradicted those of Mack et al., the disagreement is mostly based on the interpretation of mouse studies reported in Mobus et al.^11,16^. Experimentally induced mouse AD many not fully represent human disease^11,16^.

Focusing on the human subject results from the three studies, NK cell numbers differ in circulation and lesional skin in those with AD as compared to those without AD. Those with AD are more likely to have poorly educated NK cells (less active) in circulation that are more likely to express inhibitory receptors from NCR or KIR families^11,12,16^. Mack et al. and Mobus et al. both found larger numbers NK cells in human lesional skin^11,16^. Although the full functional consequences are unknown, it is possible that allelic polymorphisms such as KIR2DL5*001:01 impact KIR protein expression and function in a group of mature NK cells found in AD lesional skin. A previous study showed that specific *KIR2DL5A* alleles such as KIR2DL5*001:01 are expressed on the NK cell surface, whereas by a variety of mechanisms such as silenced transcription (e.g., KIR2DL5B*006:01) or intracellular retention (e.g., KIR2DL5A*005), some *KIR2DL5* alleles are not expressed^35^.

This is an epidemiologic study and inherently has limitations. We focused on common allelic variants, not rare variants. This was primarily due to sample size and power considerations. It is likely that less common alleles in the genes *KIR2DS1* and *KIR2DS5* are associated with AD. However, from a population perspective, we focused on alleles that are likely relevant to population at large. In addition, while rare variants might help explain disease mechanisms, common variants are critical for conducting translational studies on humans to further our understanding of the immunologic mechanisms associated with NK cell function and AD. Larger studies should be conducted to investigate less common variants. We evaluated genetic variation but did not evaluate the expression of NK surface receptors and how these receptors interact with keratinocytes, antigen presenting cells or other cells. Based on our studies, these evaluations should be done specifically for KIR2DL5*001:01 in future studies. We were also not able to evaluate NK cell numbers in circulation or in tissue. These studies are essential to further our understanding of how KIR alleles effect NK cell function with respect to AD. Finally, it is possible that our study does not generalize to other populations with AD. However, strengths of our study include that our participants were recruited from multiple centers and, unlike previous studies, we were able to assess associations in a relatively large number of African Americans.

In summary we conducted a case–control study of White and Black individuals from the United States with physician confirmed AD. We demonstrated that the common allele KIR2DL5*001:01 is associated with AD and that this effect is augmented in the presence of HLA-B*-21TT. This finding is consistent with previous tissue based reports^11^. Our study adds to the growing literature on the genetic basis of the immune dysregulation of AD and, specifically, that *KIR* allelic variation is associated with an increased risk of AD^20,27,29,40,41^. Further a paradigm shift has occurred in understanding of the immunology of AD suggesting that NK deficiency may be real, may interplay with barrier dysfunction and that treatment studies specifically examining NK induction and modulation are now warranted^11^. Specifically, studies of NK cell immunologic function in those with AD may help explain disease variability, different disease immunologic endotypes, differences in genetic predilection for AD by race, as well as treatment failure^10,42–45^.

## Materials and methods

### Study population

Study subjects were enrolled between 2015 and 2020 as participants in the *Genetics of Atopic Dermatitis* (GAD) cohort; details of this study were published earlier^30,46^. All subjects were examined and diagnosed by dermatologists with expertise in the diagnosis of AD. All subjects had a history and an exam consistent with AD (cases) or no history of AD (controls). We evaluated self-reported race. This decision was based on previous evaluations of US studies on AD of race and genetic admixture that have shown that self-reported race is highly correlated with genetically determined ancestry (also see Supplementary Table 2)^47–49^. All subjects in the GAD cohort provided informed consents and/or assents that was approved by the appropriate Institutional Review Boards including the institutional review boards from the University of Pennsylvania, Washington University of St. Louis, Pennsylvania State University, and Children’s Hospital of Philadelphia. DNA samples analyzed for this study were anonymized. All studies were conducted consistent with ethical standards and guidelines of *Scientific Reports*.

### DNA analysis

#### HLA genotyping

DNA was collected using Oragene DNA collection kits (DNA Genotek, Ottawa Canada) as previously reported^49–52^. HLA Class I genes (-A, -B, and -C) were sequenced using targeted amplicon-based NGS as previously described^50–52^.

We focused on HLA ligands known to interact with KIR genes studied. HLA-B leader sequence is defined at location − 21 (amino acids T or M) of HLA-B. The HLA-C epitope that interacts with these *KIR* genes is at position 80 and is defined by amino acids N and K (i.e., C1/C2 KIR binding site)^24,26,27,53^. Residue locations were based on IMGT data^54,55^. These data were previously published^22,30,51^.

#### KIR allelic sequencing

*KIR* allele typing, requiring 15 µl of DNA in the concentration range of 20–50 ng/µl, was conducted by the Institute for Immunology and Infectious Diseases (IIID) of Murdoch University in Perth, Western Australia. Uniquely indexed primers were designed to target *KIR* exons 3, 4, and 5 (D0, D1 and D2 Domain). The primers chosen were adapted and refined from published literature^56^. Primer mixes were prepared containing 2–4 primers per amplicon to cover all *KIR* genes in a multiplexed assay with an amplicon size ~ 400 bp. Five separate PCR reactions were setup for each sample to cover all targeted exons in 12.5 μl volume in 96-well plates with GoTaq DNA Polymerase (Promega) and the associated buffer system. As each sample was uniquely indexed during the PCR reactions, all amplicons from the PCR reactions were pooled using volumes appropriate to obtain balanced read coverage for each *KIR* exon. The minimum coverage of 10 reads and > 10% of the reads was accepted to be called an allele, however in most cases we have a read coverage of over 50 reads and majority of the heterozygous calls were larger. The products were sequenced on the Miseq using 600V3 chemistry. Post sequencing, quality filtered paired reads were demultiplexed based on unique molecular sequence for each sample and each set of overlapping paired reads were merged based on the Q30 scores leading to a single read with a median read length of up to 500bps. spanning the full amplicon. Since these amplicons span across the full exon, the SNPs within the exon were phased. These reads were then aligned to a reference sequence containing all KIR genes using CLCbio genomics workbench. Results were based on the current KIR dataset available IMGT/KIR Sequence Database—release: v2.10.0, date: 16 December 2020 (https://www.ebi.ac.uk/ipd/kir/docs/version.html). Multi mapped reads were extracted and reassigned based on set defined rules based on KIR haplotypes and heterozygosity and final assignments are reported as igroups^57^. Please refer to the link below for the list of condensed alleles in the i-groups. http://www.iiid.com.au/s/iiid_KIR_iGroups_v2100.xlsx. Each pool was then ligated with unique Illumina indexes and sequencing adapters ready for Illumina sequencing. The alignment files (bam files) were then used for assigning the *KIR* allele calls using **VGAS** which generates the cluster consensus sequences. Following alignment, using an inhouse developed application **All Class** the reads are moved to the correct gene by comparing each mapped read to the reference gene dataset generated from IMGT KIR database reads that do not map to any reference sequence data and reads mapping below the defined minimum cut off are discarded KIR alleles are presented to two fields. Validation of this pipeline has been shown by using this technique to demonstrate that an allele distribution analysis of the Perth HIV cohort dataset using this pipeline compares quite well to the frequency distribution in the United States^58^.

### Analysis

The prevalence of *KIR* genes was estimated at the subject level and are presented with 95% confidence intervals (CI). The frequencies of *KIR* alleles were estimated at the chromosome level and are also presented with 95% confidence intervals (CI). These parameters were estimated for those with and without AD and by self-reported race (White or Black) which we found to be highly correlated with genetically determined ancestry in a previous study^49^. We limited our analyses to common alleles (i.e., frequency of ≥ 0.05). The odds ratio (OR) of having AD was estimated using logistic regression. Analyses of *KIR* genes and alleles are complicated and, in prior studies, have been inconsistently presented^18,19,59,60^. Our analyses focused on three primary comparisons of *2DS5*, *2DL5*, and *2DS1* KIR genes: (1) presence or absence of the *KIR* gene, (2) the association of a KIR allele (i.e., having the gene but not the allele, heterozygote for the allele, and homozygote for the allele) to the absence of the *KIR* gene, and (3) presence or absence of a specific *KIR* allele in the whole cohort. The association of known interactions between *KIR* and HLA ligand pairs was evaluated based on the presence or absence of the HLA ligand^24,60,61^.

Models were not adjusted for other atopic illnesses like asthma, seasonal allergies or food allergies because, as previously noted by studies of the “atopic march”, these illnesses are likely on the same causal pathway^62^. Our hypotheses were determined a priori and, based on previously published data of *KIR* genes known to be associated with AD, and hence *p*-values were corrected per gene for multiple testing^22^.

All statistical analyses were conducted using Stata Version 17.0 (College Station, TX).

### Ethics statement

Human subject involvement was reviewed and approved by the Institutional Review Board of the University of Pennsylvania. All studies were conducted consistent with the ethical and regulatory requirements of *Scientific Reports*.

## Supplementary Information

Supplementary Tables.

## Data Availability

The genomic datasets can be found on NCBI SRA at the following link: https://www.ncbi.nlm.nih.gov/sra/PRJNA802230.

## Abbreviations

AF: Allelic frequency
AD: Atopic dermatitis
CI: Confidence interval
CNV: Copy number variation
DNA: Deoxyribonucleic acid
GAD: Genetics of atopic dermatitis
GWAS: Genome wide association study
HLA: Human leukocyte antigen
IMGT: International immunogenetics project
KIR: Killer cell Ig-like receptor family
MHC: Major histocompatibility complex
NCR: Natural cytotoxicity receptors
NK: Natural killer
NGS: Next generation sequencing
OR: Odds ratio
POEM: Patient oriented eczema measure
*p*: *p*-Value
