# Supplementary material for: Association of KIR2DL5, KIR2DS5, and KIR2DS1 allelic variation and atopic dermatitis
Source: Sci Rep. 2023 Jan 31;13:1730. doi: 10.1038/s41598-023-28847-y (PMC9889380; doi:10.1038/s41598-023-28847-y)
Supplement: Supplementary file 1 — Supplementary Tables. [file 41598_2023_28847_MOESM1_ESM.docx]

**Supplement in two tables:**

***Association of KIR2DL5, KIR2DS5, and KIR2DS1 allelic variation and Atopic Dermatitis***

David J Margolis MD PhD* ^1,2^, Nandita Mitra PhD^1^, Ole J Hoffstad MA^2^, Ronald Berna BA^2^, Brian S Kim MD MTR^3^, Abha Chopra PhD^4^, and Elizabeth J Phillips MD^4,5^

1. Department of Biostatistics, Epidemiology and Informatics, Perelman School of Medicine, Philadelphia, Pennsylvania

2. Department of Dermatology, Perelman School of Medicine, University of Pennsylvania, Philadelphia, Pennsylvania

3. Division of Dermatology, Department of Medicine, Center for the Study of Itch, Washington University School of Medicine, St. Louis, Missouri

4. Departments of Medicine, Vanderbilt School of Medicine, Nashville, Tennessee

5. Institute for Immunology and Infectious Diseases, Murdoch University, Murdoch, Australia

**Supplement Table 1**: Allelic frequencies (AF) for all KIR genes using the GAD cohort. The allelic frequencies are present for the full cohort and by race (White or Black). The frequencies are presented with 95% confidence intervals (CI) and with the sample size with that allele. When alleles could not be fully specified the ambiguity is presented. Alleles with AF of greater than 0.05% that were noted in KIR genes 2LD5, 2DS1, and 2DS5 are also presented in Table 1. Sheet 1- full cohort; Sheet 2-Subjects without AD (controls) ; and Sheet 3-Subjects with AD(cases)

A: Allelic frequencies for all KIR genes using the GAD cohort: Full cohort.

|  | Full Cohort | | White | | Black | |
| --- | --- | --- | --- | --- | --- | --- |
| Allele name | n | AF(95% CI) | n | AF(95% CI) | n | AF(95% CI) |
| KIR2DL1*0040101i | 139 | 0.14 (0.12,0.16) | 76 | 0.14 (0.11,0.17) | 57 | 0.15 (0.12,0.20) |
| KIR2DL1*0010101i | 244 | 0.25 (0.22,0.28) | 194 | 0.35 (0.31,0.39) | 40 | 0.11 (0.08,0.15) |
| KIR2DL1*00101i | 2 | 0.00 (0.00,0.01) | 2 | 0.00 (0.00,0.01) | 0 | 0.00 (0.00,0.01) |
| KIR2DL1*00301i | 439 | 0.45 (0.42,0.48) | 230 | 0.41 (0.37,0.45) | 177 | 0.48 (0.43,0.53) |
| KIR2DL1*00303i | 33 | 0.03 (0.02,0.05) | 14 | 0.02 (0.01,0.04) | 20 | 0.05 (0.03,0.08) |
| KIR2DL1*00404i | 5 | 0.01 (0.00,0.01) | 2 | 0.00 (0.00,0.01) | 3 | 0.01 (0.00,0.02) |
| KIR2DL1*00601i | 13 | 0.01 (0.01,0.02) | 1 | 0.00 (0.00,0.01) | 12 | 0.03 (0.02,0.06) |
| KIR2DL1*007i | 25 | 0.03 (0.02,0.04) | 8 | 0.01 (0.01,0.03) | 14 | 0.04 (0.02,0.06) |
| KIR2DL1*008i | 4 | 0.00 (0.00,0.01) | 4 | 0.01 (0.00,0.02) | 0 | 0.00 (0.00,0.01) |
| KIR2DL1*01201i | 28 | 0.03 (0.02,0.04) | 7 | 0.01 (0.01,0.03) | 21 | 0.06 (0.04,0.09) |
| KIR2DL1*013Ni | 1 | 0.00 (0.00,0.01) | 0 | 0.00 (0.00,0.01) | 1 | 0.00 (0.00,0.02) |
| KIR2DL1*014i | 4 | 0.00 (0.00,0.01) | 0 | 0.00 (0.00,0.01) | 4 | 0.01 (0.00,0.03) |
| KIR2DL1*029i | 1 | 0.00 (0.00,0.01) | 0 | 0.00 (0.00,0.01) | 1 | 0.00 (0.00,0.02) |
| KIR2DL1*0320101Ni | 4 | 0.00 (0.00,0.01) | 4 | 0.01 (0.00,0.02) | 0 | 0.00 (0.00,0.01) |
| KIR2DL1* gene present | 8 | 0.01 (0.00,0.02) | 0 | 0.00 (0.00,0.01) | 8 | 0.02 (0.01,0.04) |
| KIR2DL2*0010101i | 281 | 0.29 (0.26,0.32) | 168 | 0.30 (0.26,0.34) | 99 | 0.27 (0.22,0.32) |
| KIR2DL2*00102i | 3 | 0.00 (0.00,0.01) | 0 | 0.00 (0.00,0.01) | 3 | 0.01 (0.00,0.02) |
| KIR2DL2*0030101i | 198 | 0.20 (0.18,0.23) | 135 | 0.24 (0.21,0.28) | 55 | 0.15 (0.11,0.19) |
| KIR2DL2*004i | 7 | 0.01 (0.00,0.01) | 0 | 0.00 (0.00,0.01) | 7 | 0.02 (0.01,0.04) |
| KIR2DL2*00601i | 4 | 0.00 (0.00,0.01) | 0 | 0.00 (0.00,0.01) | 4 | 0.01 (0.00,0.03) |
| KIR2DL2*00602i | 18 | 0.02 (0.01,0.03) | 0 | 0.00 (0.00,0.01) | 18 | 0.05 (0.03,0.08) |
| KIR2DL2*00602i or 00602i 009i | 1 | 0.00 (0.00,0.01) | 1 | 0.00 (0.00,0.01) | 0 | 0.00 (0.00,0.01) |
| KIR2DL2*009i | 6 | 0.01 (0.00,0.01) | 2 | 0.00 (0.00,0.01) | 4 | 0.01 (0.00,0.03) |
| KIR2DL2*0602i | 4 | 0.00 (0.00,0.01) | 0 | 0.00 (0.00,0.01) | 4 | 0.01 (0.00,0.03) |
| KIR2DL3*0010101i | 721 | 0.73 (0.71,0.76) | 441 | 0.78 (0.75,0.82) | 237 | 0.64 (0.59,0.69) |
| KIR2DL3*00103i | 22 | 0.02 (0.01,0.03) | 7 | 0.01 (0.01,0.03) | 16 | 0.04 (0.03,0.07) |
| KIR2DL3*00110i | 5 | 0.01 (0.00,0.01) | 5 | 0.01 (0.00,0.02) | 0 | 0.00 (0.00,0.01) |
| KIR2DL3*004i | 1 | 0.00 (0.00,0.01) | 0 | 0.00 (0.00,0.01) | 1 | 0.00 (0.00,0.02) |
| KIR2DL3*00501i | 97 | 0.10 (0.08,0.12) | 44 | 0.08 (0.06,0.10) | 51 | 0.14 (0.10,0.18) |
| KIR2DL3*00502i | 2 | 0.00 (0.00,0.01) | 0 | 0.00 (0.00,0.01) | 2 | 0.01 (0.00,0.02) |
| KIR2DL3*009i | 1 | 0.00 (0.00,0.01) | 1 | 0.00 (0.00,0.01) | 0 | 0.00 (0.00,0.01) |
| KIR2DL3*011i | 4 | 0.00 (0.00,0.01) | 0 | 0.00 (0.00,0.01) | 4 | 0.01 (0.00,0.03) |
| KIR2DL3*013i | 7 | 0.01 (0.00,0.01) | 0 | 0.00 (0.00,0.01) | 7 | 0.02 (0.01,0.04) |
| KIR2DL3*014 | 2 | 0.00 (0.00,0.01) | 0 | 0.00 (0.00,0.01) | 2 | 0.01 (0.00,0.02) |
| KIR2DL3*014i | 8 | 0.01 (0.00,0.02) | 0 | 0.00 (0.00,0.01) | 8 | 0.02 (0.01,0.04) |
| KIR2DL3*015i | 1 | 0.00 (0.00,0.01) | 1 | 0.00 (0.00,0.01) | 0 | 0.00 (0.00,0.01) |
| KIR2DL3*033i | 23 | 0.02 (0.01,0.03) | 21 | 0.04 (0.02,0.06) | 2 | 0.01 (0.00,0.02) |
| KIR2DL3* gene present | 6 | 0.01 (0.00,0.01) | 0 | 0.00 (0.00,0.01) | 4 | 0.01 (0.00,0.03) |
| KIR2DL4*0010201i | 356 | 0.36 (0.33,0.39) | 169 | 0.30 (0.26,0.34) | 165 | 0.45 (0.40,0.50) |
| KIR2DL4*00201i | 130 | 0.13 (0.11,0.16) | 86 | 0.15 (0.12,0.19) | 44 | 0.12 (0.09,0.16) |
| KIR2DL4*00202i | 150 | 0.15 (0.13,0.18) | 91 | 0.16 (0.13,0.20) | 51 | 0.14 (0.10,0.18) |
| KIR2DL4*0050101i | 285 | 0.29 (0.26,0.32) | 194 | 0.35 (0.31,0.39) | 73 | 0.20 (0.16,0.24) |
| KIR2DL4*00601i | 42 | 0.04 (0.03,0.06) | 19 | 0.03 (0.02,0.05) | 19 | 0.05 (0.03,0.08) |
| KIR2DL4*010i | 7 | 0.01 (0.00,0.01) | 0 | 0.00 (0.00,0.01) | 7 | 0.02 (0.01,0.04) |
| KIR2DL4*017i | 1 | 0.00 (0.00,0.01) | 0 | 0.00 (0.00,0.01) | 1 | 0.00 (0.00,0.02) |
| KIR2DL4*018i | 1 | 0.00 (0.00,0.01) | 0 | 0.00 (0.00,0.01) | 1 | 0.00 (0.00,0.02) |
| KIR2DL4*021i | 2 | 0.00 (0.00,0.01) | 0 | 0.00 (0.00,0.01) | 2 | 0.01 (0.00,0.02) |
| KIR2DL4*023i | 6 | 0.01 (0.00,0.01) | 1 | 0.00 (0.00,0.01) | 5 | 0.01 (0.00,0.03) |
| KIR2DL5*0020101i | 224 | 0.23 (0.20,0.26) | 137 | 0.24 (0.21,0.28) | 71 | 0.19 (0.15,0.24) |
| KIR2DL5*0010101i | 277 | 0.28 (0.25,0.31) | 145 | 0.26 (0.22,0.30) | 115 | 0.31 (0.27,0.36) |
| KIR2DL5*00103i | 1 | 0.00 (0.00,0.01) | 1 | 0.00 (0.00,0.01) | 0 | 0.00 (0.00,0.01) |
| KIR2DL5*00203i | 5 | 0.01 (0.00,0.01) | 1 | 0.00 (0.00,0.01) | 3 | 0.01 (0.00,0.02) |
| KIR2DL5*003i | 4 | 0.00 (0.00,0.01) | 0 | 0.00 (0.00,0.01) | 4 | 0.01 (0.00,0.03) |
| KIR2DL5*004i | 5 | 0.01 (0.00,0.01) | 0 | 0.00 (0.00,0.01) | 5 | 0.01 (0.00,0.03) |
| KIR2DP1*0010201i | 127 | 0.13 (0.11,0.15) | 82 | 0.15 (0.12,0.18) | 36 | 0.10 (0.07,0.13) |
| KIR2DP1*00101i | 13 | 0.01 (0.01,0.02) | 11 | 0.02 (0.01,0.03) | 1 | 0.00 (0.00,0.02) |
| KIR2DP1*00103i | 9 | 0.01 (0.00,0.02) | 1 | 0.00 (0.00,0.01) | 8 | 0.02 (0.01,0.04) |
| KIR2DP1*0020101i | 395 | 0.40 (0.37,0.43) | 199 | 0.35 (0.31,0.40) | 171 | 0.46 (0.41,0.52) |
| KIR2DP1*00204i | 2 | 0.00 (0.00,0.01) | 2 | 0.00 (0.00,0.01) | 0 | 0.00 (0.00,0.01) |
| KIR2DP1*0030101i | 265 | 0.27 (0.24,0.30) | 199 | 0.35 (0.31,0.40) | 51 | 0.14 (0.10,0.18) |
| KIR2DP1*004i | 11 | 0.01 (0.01,0.02) | 10 | 0.02 (0.01,0.03) | 1 | 0.00 (0.00,0.02) |
| KIR2DP1*007i | 19 | 0.02 (0.01,0.03) | 5 | 0.01 (0.00,0.02) | 15 | 0.04 (0.02,0.07) |
| KIR2DP1*008i | 7 | 0.01 (0.00,0.01) | 7 | 0.01 (0.01,0.03) | 0 | 0.00 (0.00,0.01) |
| KIR2DP1*009i | 12 | 0.01 (0.01,0.02) | 0 | 0.00 (0.00,0.01) | 12 | 0.03 (0.02,0.06) |
| KIR2DP1*010i | 35 | 0.04 (0.02,0.05) | 4 | 0.01 (0.00,0.02) | 31 | 0.08 (0.06,0.12) |
| KIR2DP1*013i | 18 | 0.02 (0.01,0.03) | 1 | 0.00 (0.00,0.01) | 17 | 0.05 (0.03,0.07) |
| KIR2DP1*015i | 1 | 0.00 (0.00,0.01) | 0 | 0.00 (0.00,0.01) | 1 | 0.00 (0.00,0.02) |
| KIR2DP1*017i | 7 | 0.01 (0.00,0.01) | 7 | 0.01 (0.01,0.03) | 0 | 0.00 (0.00,0.01) |
| KIR2DP1*019i | 1 | 0.00 (0.00,0.01) | 0 | 0.00 (0.00,0.01) | 0 | 0.00 (0.00,0.01) |
| KIR2DP1* gene present | 18 | 0.02 (0.01,0.03) | 8 | 0.01 (0.01,0.03) | 10 | 0.03 (0.01,0.05) |
| KIR2DS1*0020101i | 308 | 0.31 (0.28,0.34) | 209 | 0.37 (0.33,0.41) | 73 | 0.20 (0.16,0.24) |
| KIR2DS1*004i | 9 | 0.01 (0.00,0.02) | 0 | 0.00 (0.00,0.01) | 9 | 0.02 (0.01,0.05) |
| KIR2DS1*00501i | 5 | 0.01 (0.00,0.01) | 3 | 0.01 (0.00,0.02) | 2 | 0.01 (0.00,0.02) |
| KIR2DS1* gene present | 8 | 0.01 (0.00,0.02) | 2 | 0.00 (0.00,0.01) | 6 | 0.02 (0.01,0.04) |
| KIR2DS2*0010101i | 499 | 0.51 (0.48,0.54) | 306 | 0.54 (0.50,0.59) | 171 | 0.46 (0.41,0.52) |
| KIR2DS2*00104i | 2 | 0.00 (0.00,0.01) | 0 | 0.00 (0.00,0.01) | 2 | 0.01 (0.00,0.02) |
| KIR2DS2*0010701i | 1 | 0.00 (0.00,0.01) | 0 | 0.00 (0.00,0.01) | 1 | 0.00 (0.00,0.02) |
| KIR2DS2*016i | 2 | 0.00 (0.00,0.01) | 0 | 0.00 (0.00,0.01) | 2 | 0.01 (0.00,0.02) |
| KIR2DS2* gene present | 2 | 0.00 (0.00,0.01) | 0 | 0.00 (0.00,0.01) | 2 | 0.01 (0.00,0.02) |
| KIR2DS3*00101i | 257 | 0.26 (0.23,0.29) | 159 | 0.28 (0.25,0.32) | 80 | 0.22 (0.18,0.26) |
| KIR2DS3*0030101Ni | 5 | 0.01 (0.00,0.01) | 5 | 0.01 (0.00,0.02) | 0 | 0.00 (0.00,0.01) |
| KIR2DS3*008i | 2 | 0.00 (0.00,0.01) | 2 | 0.00 (0.00,0.01) | 0 | 0.00 (0.00,0.01) |
| KIR2DS3*015i | 2 | 0.00 (0.00,0.01) | 2 | 0.00 (0.00,0.01) | 0 | 0.00 (0.00,0.01) |
| KIR2DS3*017i | 2 | 0.00 (0.00,0.01) | 0 | 0.00 (0.00,0.01) | 0 | 0.00 (0.00,0.01) |
| KIR2DS3*0210101i | 2 | 0.00 (0.00,0.01) | 0 | 0.00 (0.00,0.01) | 2 | 0.01 (0.00,0.02) |
| KIR2DS3*0220101i | 2 | 0.00 (0.00,0.01) | 2 | 0.00 (0.00,0.01) | 0 | 0.00 (0.00,0.01) |
| KIR2DS4*0010101i | 370 | 0.38 (0.35,0.41) | 170 | 0.30 (0.26,0.34) | 173 | 0.47 (0.42,0.52) |
| KIR2DS4*0030101i | 217 | 0.22 (0.20,0.25) | 151 | 0.27 (0.23,0.31) | 53 | 0.14 (0.11,0.18) |
| KIR2DS4*0040101i | 56 | 0.06 (0.04,0.07) | 23 | 0.04 (0.03,0.06) | 29 | 0.08 (0.05,0.11) |
| KIR2DS4*0060101i | 162 | 0.16 (0.14,0.19) | 100 | 0.18 (0.15,0.21) | 62 | 0.17 (0.13,0.21) |
| KIR2DS4*009i | 12 | 0.01 (0.01,0.02) | 0 | 0.00 (0.00,0.01) | 12 | 0.03 (0.02,0.06) |
| KIR2DS4*010i | 89 | 0.09 (0.07,0.11) | 69 | 0.12 (0.10,0.15) | 18 | 0.05 (0.03,0.08) |
| KIR2DS4*010i or 0030101i 016i | 29 | 0.03 (0.02,0.04) | 21 | 0.04 (0.02,0.06) | 6 | 0.02 (0.01,0.04) |
| KIR2DS4*012i | 3 | 0.00 (0.00,0.01) | 0 | 0.00 (0.00,0.01) | 3 | 0.01 (0.00,0.02) |
| KIR2DS4*013i | 2 | 0.00 (0.00,0.01) | 0 | 0.00 (0.00,0.01) | 2 | 0.01 (0.00,0.02) |
| KIR2DS5*0020101i | 244 | 0.25 (0.22,0.28) | 172 | 0.31 (0.27,0.35) | 54 | 0.15 (0.11,0.19) |
| KIR2DS5*003i | 8 | 0.01 (0.00,0.02) | 0 | 0.00 (0.00,0.01) | 8 | 0.02 (0.01,0.04) |
| KIR2DS5*00501i | 31 | 0.03 (0.02,0.04) | 2 | 0.00 (0.00,0.01) | 29 | 0.08 (0.05,0.11) |
| KIR2DS5*00502i | 3 | 0.00 (0.00,0.01) | 0 | 0.00 (0.00,0.01) | 3 | 0.01 (0.00,0.02) |
| KIR2DS5*0060101i | 9 | 0.01 (0.00,0.02) | 0 | 0.00 (0.00,0.01) | 9 | 0.02 (0.01,0.05) |
| KIR2DS5*009i | 19 | 0.02 (0.01,0.03) | 2 | 0.00 (0.00,0.01) | 17 | 0.05 (0.03,0.07) |
| KIR2DS5*010i | 2 | 0.00 (0.00,0.01) | 0 | 0.00 (0.00,0.01) | 2 | 0.01 (0.00,0.02) |
| KIR2DS5*011i | 4 | 0.00 (0.00,0.01) | 0 | 0.00 (0.00,0.01) | 4 | 0.01 (0.00,0.03) |
| KIR2DS5* gene present | 4 | 0.00 (0.00,0.01) | 0 | 0.00 (0.00,0.01) | 4 | 0.01 (0.00,0.03) |
| KIR3DL1*0150201i | 147 | 0.15 (0.13,0.17) | 70 | 0.12 (0.10,0.15) | 58 | 0.16 (0.12,0.20) |
| KIR3DL1*0200101i | 35 | 0.04 (0.02,0.05) | 14 | 0.02 (0.01,0.04) | 18 | 0.05 (0.03,0.08) |
| KIR3DL1*0010101i | 156 | 0.16 (0.14,0.18) | 105 | 0.19 (0.16,0.22) | 42 | 0.11 (0.08,0.15) |
| KIR3DL1*0020101i | 92 | 0.09 (0.08,0.11) | 74 | 0.13 (0.10,0.16) | 15 | 0.04 (0.02,0.07) |
| KIR3DL1*0040101i | 134 | 0.14 (0.12,0.16) | 91 | 0.16 (0.13,0.20) | 43 | 0.12 (0.09,0.15) |
| KIR3DL1*0050101i | 112 | 0.11 (0.09,0.14) | 84 | 0.15 (0.12,0.18) | 24 | 0.07 (0.04,0.10) |
| KIR3DL1*0070101i | 142 | 0.14 (0.12,0.17) | 34 | 0.06 (0.04,0.08) | 102 | 0.28 (0.23,0.33) |
| KIR3DL1*0080101i | 40 | 0.04 (0.03,0.06) | 34 | 0.06 (0.04,0.08) | 3 | 0.01 (0.00,0.02) |
| KIR3DL1*0090101i | 14 | 0.01 (0.01,0.02) | 11 | 0.02 (0.01,0.03) | 3 | 0.01 (0.00,0.02) |
| KIR3DL1*0090101i or 0070101i 054i | 1 | 0.00 (0.00,0.01) | 0 | 0.00 (0.00,0.01) | 0 | 0.00 (0.00,0.01) |
| KIR3DL1*019i | 8 | 0.01 (0.00,0.02) | 8 | 0.01 (0.01,0.03) | 0 | 0.00 (0.00,0.01) |
| KIR3DL1*022i | 12 | 0.01 (0.01,0.02) | 0 | 0.00 (0.00,0.01) | 12 | 0.03 (0.02,0.06) |
| KIR3DL1*023i | 1 | 0.00 (0.00,0.01) | 0 | 0.00 (0.00,0.01) | 1 | 0.00 (0.00,0.02) |
| KIR3DL1*028i | 5 | 0.01 (0.00,0.01) | 0 | 0.00 (0.00,0.01) | 5 | 0.01 (0.00,0.03) |
| KIR3DL1*0290101i | 2 | 0.00 (0.00,0.01) | 2 | 0.00 (0.00,0.01) | 0 | 0.00 (0.00,0.01) |
| KIR3DL1*033i | 12 | 0.01 (0.01,0.02) | 0 | 0.00 (0.00,0.01) | 12 | 0.03 (0.02,0.06) |
| KIR3DL1*03501i | 1 | 0.00 (0.00,0.01) | 0 | 0.00 (0.00,0.01) | 1 | 0.00 (0.00,0.02) |
| KIR3DL1*042i | 3 | 0.00 (0.00,0.01) | 3 | 0.01 (0.00,0.02) | 0 | 0.00 (0.00,0.01) |
| KIR3DL1*053i | 5 | 0.01 (0.00,0.01) | 3 | 0.01 (0.00,0.02) | 2 | 0.01 (0.00,0.02) |
| KIR3DL1*05901i | 10 | 0.01 (0.00,0.02) | 0 | 0.00 (0.00,0.01) | 10 | 0.03 (0.01,0.05) |
| KIR3DL1*061i | 1 | 0.00 (0.00,0.01) | 0 | 0.00 (0.00,0.01) | 1 | 0.00 (0.00,0.02) |
| KIR3DL1*063i | 2 | 0.00 (0.00,0.01) | 0 | 0.00 (0.00,0.01) | 2 | 0.01 (0.00,0.02) |
| KIR3DL1*065i | 1 | 0.00 (0.00,0.01) | 0 | 0.00 (0.00,0.01) | 1 | 0.00 (0.00,0.02) |
| KIR3DL1*072i | 1 | 0.00 (0.00,0.01) | 1 | 0.00 (0.00,0.01) | 0 | 0.00 (0.00,0.01) |
| KIR3DL1*089i | 1 | 0.00 (0.00,0.01) | 0 | 0.00 (0.00,0.01) | 1 | 0.00 (0.00,0.02) |
| KIR3DL1*090101i | 1 | 0.00 (0.00,0.01) | 0 | 0.00 (0.00,0.01) | 1 | 0.00 (0.00,0.02) |
| KIR3DL1*092i | 1 | 0.00 (0.00,0.01) | 0 | 0.00 (0.00,0.01) | 1 | 0.00 (0.00,0.02) |
| KIR3DL2*0010101i | 352 | 0.36 (0.33,0.39) | 194 | 0.35 (0.31,0.39) | 139 | 0.38 (0.33,0.43) |
| KIR3DL2*0020101i | 119 | 0.12 (0.10,0.14) | 84 | 0.15 (0.12,0.18) | 25 | 0.07 (0.04,0.10) |
| KIR3DL2*00102i | 1 | 0.00 (0.00,0.01) | 1 | 0.00 (0.00,0.01) | 0 | 0.00 (0.00,0.01) |
| KIR3DL2*00301i | 71 | 0.07 (0.06,0.09) | 44 | 0.08 (0.06,0.10) | 27 | 0.07 (0.05,0.10) |
| KIR3DL2*00302i | 17 | 0.02 (0.01,0.03) | 0 | 0.00 (0.00,0.01) | 17 | 0.05 (0.03,0.07) |
| KIR3DL2*00501i | 24 | 0.02 (0.02,0.04) | 21 | 0.04 (0.02,0.06) | 3 | 0.01 (0.00,0.02) |
| KIR3DL2*00501i or 00301i 023i | 19 | 0.02 (0.01,0.03) | 17 | 0.03 (0.02,0.05) | 2 | 0.01 (0.00,0.02) |
| KIR3DL2*00501i or 023i 026i | 5 | 0.01 (0.00,0.01) | 5 | 0.01 (0.00,0.02) | 0 | 0.00 (0.00,0.01) |
| KIR3DL2*00601i | 110 | 0.11 (0.09,0.13) | 65 | 0.12 (0.09,0.15) | 39 | 0.11 (0.08,0.14) |
| KIR3DL2*008i | 20 | 0.02 (0.01,0.03) | 8 | 0.01 (0.01,0.03) | 10 | 0.03 (0.01,0.05) |
| KIR3DL2*008i or 016i 021i | 17 | 0.02 (0.01,0.03) | 9 | 0.02 (0.01,0.03) | 6 | 0.02 (0.01,0.04) |
| KIR3DL2*008i or 016i 027i | 7 | 0.01 (0.00,0.01) | 1 | 0.00 (0.00,0.01) | 6 | 0.02 (0.01,0.04) |
| KIR3DL2*0090101i | 1 | 0.00 (0.00,0.01) | 0 | 0.00 (0.00,0.01) | 0 | 0.00 (0.00,0.01) |
| KIR3DL2*00901i | 39 | 0.04 (0.03,0.05) | 27 | 0.05 (0.03,0.07) | 10 | 0.03 (0.01,0.05) |
| KIR3DL2*00901i or 021i 036i | 1 | 0.00 (0.00,0.01) | 1 | 0.00 (0.00,0.01) | 0 | 0.00 (0.00,0.01) |
| KIR3DL2*00901i or 016i 023i | 4 | 0.00 (0.00,0.01) | 3 | 0.01 (0.00,0.02) | 1 | 0.00 (0.00,0.02) |
| KIR3DL2*012i | 1 | 0.00 (0.00,0.01) | 1 | 0.00 (0.00,0.01) | 0 | 0.00 (0.00,0.01) |
| KIR3DL2*01301i | 41 | 0.04 (0.03,0.06) | 2 | 0.00 (0.00,0.01) | 39 | 0.11 (0.08,0.14) |
| KIR3DL2*015i or 0020101i 00601i | 28 | 0.03 (0.02,0.04) | 22 | 0.04 (0.02,0.06) | 1 | 0.00 (0.00,0.02) |
| KIR3DL2*018i | 11 | 0.01 (0.01,0.02) | 10 | 0.02 (0.01,0.03) | 1 | 0.00 (0.00,0.02) |
| KIR3DL2*01901i | 3 | 0.00 (0.00,0.01) | 1 | 0.00 (0.00,0.01) | 2 | 0.01 (0.00,0.02) |
| KIR3DL2*01901i or 00102i 112i or 00601i 00901i | 10 | 0.01 (0.00,0.02) | 6 | 0.01 (0.00,0.02) | 3 | 0.01 (0.00,0.02) |
| KIR3DL2*01901i or 00601i 06201i | 1 | 0.00 (0.00,0.01) | 1 | 0.00 (0.00,0.01) | 0 | 0.00 (0.00,0.01) |
| KIR3DL2*019 01i or 00901i 015i | 3 | 0.00 (0.00,0.01) | 1 | 0.00 (0.00,0.01) | 2 | 0.01 (0.00,0.02) |
| KIR3DL2*01901i or 027i 036i | 1 | 0.00 (0.00,0.01) | 1 | 0.00 (0.00,0.01) | 0 | 0.00 (0.00,0.01) |
| KIR3DL2*020i | 1 | 0.00 (0.00,0.01) | 0 | 0.00 (0.00,0.01) | 1 | 0.00 (0.00,0.02) |
| KIR3DL2*021i | 1 | 0.00 (0.00,0.01) | 1 | 0.00 (0.00,0.01) | 0 | 0.00 (0.00,0.01) |
| KIR3DL2*023i | 5 | 0.01 (0.00,0.01) | 0 | 0.00 (0.00,0.01) | 5 | 0.01 (0.00,0.03) |
| KIR3DL2*025i or 00501i 01301i | 1 | 0.00 (0.00,0.01) | 1 | 0.00 (0.00,0.01) | 0 | 0.00 (0.00,0.01) |
| KIR3DL2*025i or 01301i 023i | 1 | 0.00 (0.00,0.01) | 0 | 0.00 (0.00,0.01) | 1 | 0.00 (0.00,0.02) |
| KIR3DL2*026i or 0020101i 00301i | 17 | 0.02 (0.01,0.03) | 15 | 0.03 (0.02,0.04) | 1 | 0.00 (0.00,0.02) |
| KIR3DL2*027i | 1 | 0.00 (0.00,0.01) | 1 | 0.00 (0.00,0.01) | 0 | 0.00 (0.00,0.01) |
| KIR3DL2*027i or 00601i 021i | 1 | 0.00 (0.00,0.01) | 1 | 0.00 (0.00,0.01) | 0 | 0.00 (0.00,0.01) |
| KIR3DL2*029i | 6 | 0.01 (0.00,0.01) | 1 | 0.00 (0.00,0.01) | 5 | 0.01 (0.00,0.03) |
| KIR3DL2*032i | 1 | 0.00 (0.00,0.01) | 0 | 0.00 (0.00,0.01) | 1 | 0.00 (0.00,0.02) |
| KIR3DL2*033i | 1 | 0.00 (0.00,0.01) | 0 | 0.00 (0.00,0.01) | 1 | 0.00 (0.00,0.02) |
| KIR3DL2*034i | 2 | 0.00 (0.00,0.01) | 2 | 0.00 (0.00,0.01) | 0 | 0.00 (0.00,0.01) |
| KIR3DL2*037i or 00901i | 1 | 0.00 (0.00,0.01) | 0 | 0.00 (0.00,0.01) | 0 | 0.00 (0.00,0.01) |
| KIR3DL2*037i or 01301i | 2 | 0.00 (0.00,0.01) | 1 | 0.00 (0.00,0.01) | 1 | 0.00 (0.00,0.02) |
| KIR3DL2*039i | 1 | 0.00 (0.00,0.01) | 0 | 0.00 (0.00,0.01) | 1 | 0.00 (0.00,0.02) |
| KIR3DL2*040i | 4 | 0.00 (0.00,0.01) | 1 | 0.00 (0.00,0.01) | 3 | 0.01 (0.00,0.02) |
| KIR3DL2*04301i or 0050 | 8 | 0.01 (0.00,0.02) | 7 | 0.01 (0.01,0.03) | 1 | 0.00 (0.00,0.02) |
| KIR3DL2*04301i or 0060 | 1 | 0.00 (0.00,0.01) | 0 | 0.00 (0.00,0.01) | 1 | 0.00 (0.00,0.02) |
| KIR3DL2*04301i or 015i | 1 | 0.00 (0.00,0.01) | 1 | 0.00 (0.00,0.01) | 0 | 0.00 (0.00,0.01) |
| KIR3DL2*051i | 3 | 0.00 (0.00,0.01) | 1 | 0.00 (0.00,0.01) | 2 | 0.01 (0.00,0.02) |
| KIR3DL2*059ior023i02 | 1 | 0.00 (0.00,0.01) | 0 | 0.00 (0.00,0.01) | 1 | 0.00 (0.00,0.02) |
| KIR3DL2*06201i | 2 | 0.00 (0.00,0.01) | 1 | 0.00 (0.00,0.01) | 1 | 0.00 (0.00,0.02) |
| KIR3DL2*06201i or 0030 | 2 | 0.00 (0.00,0.01) | 1 | 0.00 (0.00,0.01) | 1 | 0.00 (0.00,0.02) |
| KIR3DL2*064i | 1 | 0.00 (0.00,0.01) | 0 | 0.00 (0.00,0.01) | 0 | 0.00 (0.00,0.01) |
| KIR3DL2*080i | 1 | 0.00 (0.00,0.01) | 0 | 0.00 (0.00,0.01) | 1 | 0.00 (0.00,0.02) |
| KIR3DL2*082i | 1 | 0.00 (0.00,0.01) | 1 | 0.00 (0.00,0.01) | 0 | 0.00 (0.00,0.01) |
| KIR3DL2*083i | 1 | 0.00 (0.00,0.01) | 1 | 0.00 (0.00,0.01) | 0 | 0.00 (0.00,0.01) |
| KIR3DL2*095i | 2 | 0.00 (0.00,0.01) | 0 | 0.00 (0.00,0.01) | 2 | 0.01 (0.00,0.02) |
| KIR3DL2*103i or 002010 | 3 | 0.00 (0.00,0.01) | 0 | 0.00 (0.00,0.01) | 2 | 0.01 (0.00,0.02) |
| KIR3DL2*1301i or 00201 | 1 | 0.00 (0.00,0.01) | 0 | 0.00 (0.00,0.01) | 1 | 0.00 (0.00,0.02) |
| KIR3DL3*0020201i | 119 | 0.12 (0.10,0.14) | 87 | 0.15 (0.13,0.19) | 28 | 0.08 (0.05,0.11) |
| KIR3DL3*0040201i | 32 | 0.03 (0.02,0.05) | 10 | 0.02 (0.01,0.03) | 18 | 0.05 (0.03,0.08) |
| KIR3DL3*0010101i | 367 | 0.37 (0.34,0.40) | 245 | 0.44 (0.39,0.48) | 104 | 0.28 (0.24,0.33) |
| KIR3DL3*00103i | 11 | 0.01 (0.01,0.02) | 6 | 0.01 (0.00,0.02) | 4 | 0.01 (0.00,0.03) |
| KIR3DL3*00104i | 1 | 0.00 (0.00,0.01) | 1 | 0.00 (0.00,0.01) | 0 | 0.00 (0.00,0.01) |
| KIR3DL3*0020101i | 25 | 0.03 (0.02,0.04) | 19 | 0.03 (0.02,0.05) | 6 | 0.02 (0.01,0.04) |
| KIR3DL3*0020201i or 00211i 01308i | 5 | 0.01 (0.00,0.01) | 5 | 0.01 (0.00,0.02) | 0 | 0.00 (0.00,0.01) |
| KIR3DL3*00204i | 12 | 0.01 (0.01,0.02) | 1 | 0.00 (0.00,0.01) | 11 | 0.03 (0.02,0.05) |
| KIR3DL3*0020601i | 18 | 0.02 (0.01,0.03) | 16 | 0.03 (0.02,0.05) | 2 | 0.01 (0.00,0.02) |
| KIR3DL3*0020601i or 00903i 01308i | 12 | 0.01 (0.01,0.02) | 8 | 0.01 (0.01,0.03) | 4 | 0.01 (0.00,0.03) |
| KIR3DL3*0020701i | 28 | 0.03 (0.02,0.04) | 12 | 0.02 (0.01,0.04) | 15 | 0.04 (0.02,0.07) |
| KIR3DL3*00208i | 20 | 0.02 (0.01,0.03) | 2 | 0.00 (0.00,0.01) | 17 | 0.05 (0.03,0.07) |
| KIR3DL3*00210i | 2 | 0.00 (0.00,0.01) | 1 | 0.00 (0.00,0.01) | 0 | 0.00 (0.00,0.01) |
| KIR3DL3*00211i | 2 | 0.00 (0.00,0.01) | 0 | 0.00 (0.00,0.01) | 2 | 0.01 (0.00,0.02) |
| KIR3DL3*0040201i or 0020101i 066i | 16 | 0.02 (0.01,0.03) | 7 | 0.01 (0.01,0.03) | 8 | 0.02 (0.01,0.04) |
| KIR3DL3*005i | 54 | 0.05 (0.04,0.07) | 2 | 0.00 (0.00,0.01) | 51 | 0.14 (0.10,0.18) |
| KIR3DL3*0060101i | 31 | 0.03 (0.02,0.04) | 24 | 0.04 (0.03,0.06) | 4 | 0.01 (0.00,0.03) |
| KIR3DL3*0060101i or 02102i 023i | 17 | 0.02 (0.01,0.03) | 16 | 0.03 (0.02,0.05) | 1 | 0.00 (0.00,0.02) |
| KIR3DL3*0070101i | 29 | 0.03 (0.02,0.04) | 22 | 0.04 (0.02,0.06) | 5 | 0.01 (0.00,0.03) |
| KIR3DL3*00903i | 2 | 0.00 (0.00,0.01) | 0 | 0.00 (0.00,0.01) | 2 | 0.01 (0.00,0.02) |
| KIR3DL3*00905i | 3 | 0.00 (0.00,0.01) | 0 | 0.00 (0.00,0.01) | 3 | 0.01 (0.00,0.02) |
| KIR3DL3*01001i | 47 | 0.05 (0.04,0.06) | 24 | 0.04 (0.03,0.06) | 14 | 0.04 (0.02,0.06) |
| KIR3DL3*01003i | 1 | 0.00 (0.00,0.01) | 0 | 0.00 (0.00,0.01) | 0 | 0.00 (0.00,0.01) |
| KIR3DL3*01004i | 1 | 0.00 (0.00,0.01) | 1 | 0.00 (0.00,0.01) | 0 | 0.00 (0.00,0.01) |
| KIR3DL3*01101i | 31 | 0.03 (0.02,0.04) | 18 | 0.03 (0.02,0.05) | 12 | 0.03 (0.02,0.06) |
| KIR3DL3*01101i or 012i 02102i | 5 | 0.01 (0.00,0.01) | 5 | 0.01 (0.00,0.02) | 0 | 0.00 (0.00,0.01) |
| KIR3DL3*012i | 15 | 0.02 (0.01,0.03) | 2 | 0.00 (0.00,0.01) | 13 | 0.04 (0.02,0.06) |
| KIR3DL3*012i or 1101i 023i | 4 | 0.00 (0.00,0.01) | 0 | 0.00 (0.00,0.01) | 3 | 0.01 (0.00,0.02) |
| KIR3DL3*01308i | 4 | 0.00 (0.00,0.01) | 3 | 0.01 (0.00,0.02) | 1 | 0.00 (0.00,0.02) |
| KIR3DL3*01408i | 2 | 0.00 (0.00,0.01) | 0 | 0.00 (0.00,0.01) | 2 | 0.01 (0.00,0.02) |
| KIR3DL3*01410i | 1 | 0.00 (0.00,0.01) | 1 | 0.00 (0.00,0.01) | 0 | 0.00 (0.00,0.01) |
| KIR3DL3*01413i | 1 | 0.00 (0.00,0.01) | 1 | 0.00 (0.00,0.01) | 0 | 0.00 (0.00,0.01) |
| KIR3DL3*01502i | 13 | 0.01 (0.01,0.02) | 0 | 0.00 (0.00,0.01) | 10 | 0.03 (0.01,0.05) |
| KIR3DL3*01502i or 012i 01801i | 1 | 0.00 (0.00,0.01) | 0 | 0.00 (0.00,0.01) | 1 | 0.00 (0.00,0.02) |
| KIR3DL3*01502i or 01801i 023i | 2 | 0.00 (0.00,0.01) | 0 | 0.00 (0.00,0.01) | 2 | 0.01 (0.00,0.02) |
| KIR3DL3*01801i | 5 | 0.01 (0.00,0.01) | 4 | 0.01 (0.00,0.02) | 1 | 0.00 (0.00,0.02) |
| IRX3DL301801i or 01502i 02102i | 1 | 0.00 (0.00,0.01) | 1 | 0.00 (0.00,0.01) | 0 | 0.00 (0.00,0.01) |
| KIR3DL3*032i | 5 | 0.01 (0.00,0.01) | 1 | 0.00 (0.00,0.01) | 4 | 0.01 (0.00,0.03) |
| KIR3DL3*037i | 1 | 0.00 (0.00,0.01) | 0 | 0.00 (0.00,0.01) | 1 | 0.00 (0.00,0.02) |
| KIR3DL3*040i | 15 | 0.02 (0.01,0.03) | 4 | 0.01 (0.00,0.02) | 11 | 0.03 (0.02,0.05) |
| KIR3DL3*044i | 2 | 0.00 (0.00,0.01) | 2 | 0.00 (0.00,0.01) | 0 | 0.00 (0.00,0.01) |
| KIR3DL3*045i | 3 | 0.00 (0.00,0.01) | 2 | 0.00 (0.00,0.01) | 1 | 0.00 (0.00,0.02) |
| KIR3DL3*052i | 2 | 0.00 (0.00,0.01) | 0 | 0.00 (0.00,0.01) | 2 | 0.01 (0.00,0.02) |
| KIR3DL3*057i or 0040201i 01101i | 3 | 0.00 (0.00,0.01) | 1 | 0.00 (0.00,0.01) | 2 | 0.01 (0.00,0.02) |
| KIR3DL3*057i or 066i 069i | 1 | 0.00 (0.00,0.01) | 0 | 0.00 (0.00,0.01) | 1 | 0.00 (0.00,0.02) |
| KIR3DL3*058i | 1 | 0.00 (0.00,0.01) | 0 | 0.00 (0.00,0.01) | 1 | 0.00 (0.00,0.02) |
| KIR3DL3*069i or 01101i 052i | 1 | 0.00 (0.00,0.01) | 0 | 0.00 (0.00,0.01) | 1 | 0.00 (0.00,0.02) |
| KIR3DL3*074i | 2 | 0.00 (0.00,0.01) | 2 | 0.00 (0.00,0.01) | 0 | 0.00 (0.00,0.01) |
| KIR3DL3*080Ni | 2 | 0.00 (0.00,0.01) | 2 | 0.00 (0.00,0.01) | 0 | 0.00 (0.00,0.01) |
| KIR3DL3*095i | 1 | 0.00 (0.00,0.01) | 1 | 0.00 (0.00,0.01) | 0 | 0.00 (0.00,0.01) |
| KIR3DL3*10701i or 00103i 102i | 1 | 0.00 (0.00,0.01) | 1 | 0.00 (0.00,0.01) | 0 | 0.00 (0.00,0.01) |
| KIR3DP1*0030101i | 158 | 0.16 (0.14,0.19) | 84 | 0.15 (0.12,0.18) | 65 | 0.18 (0.14,0.22) |
| KIR3DP1*001i | 580 | 0.59 (0.56,0.62) | 289 | 0.51 (0.47,0.56) | 257 | 0.70 (0.65,0.74) |
| KIR3DP1*00303i | 1 | 0.00 (0.00,0.01) | 1 | 0.00 (0.00,0.01) | 0 | 0.00 (0.00,0.01) |
| KIR3DP1*005i | 193 | 0.20 (0.17,0.22) | 154 | 0.27 (0.24,0.31) | 33 | 0.09 (0.06,0.12) |
| KIR3DP1*00604i | 3 | 0.00 (0.00,0.01) | 2 | 0.00 (0.00,0.01) | 0 | 0.00 (0.00,0.01) |
| KIR3DP1*007i | 3 | 0.00 (0.00,0.01) | 0 | 0.00 (0.00,0.01) | 3 | 0.01 (0.00,0.02) |
| KIR3DP1*008i | 3 | 0.00 (0.00,0.01) | 0 | 0.00 (0.00,0.01) | 3 | 0.01 (0.00,0.02) |
| KIR3DP1*00902i | 1 | 0.00 (0.00,0.01) | 0 | 0.00 (0.00,0.01) | 1 | 0.00 (0.00,0.02) |
| KIR3DP1*0140101i | 28 | 0.03 (0.02,0.04) | 25 | 0.04 (0.03,0.06) | 3 | 0.01 (0.00,0.02) |
| KIR3DP1*017i | 3 | 0.00 (0.00,0.01) | 0 | 0.00 (0.00,0.01) | 3 | 0.01 (0.00,0.02) |
| KIR3DP1*023i | 1 | 0.00 (0.00,0.01) | 1 | 0.00 (0.00,0.01) | 0 | 0.00 (0.00,0.01) |
| KIR3DP1*029i | 2 | 0.00 (0.00,0.01) | 0 | 0.00 (0.00,0.01) | 0 | 0.00 (0.00,0.01) |
| KIR3DS1*010i | 272 | 0.28 (0.25,0.31) | 190 | 0.34 (0.30,0.38) | 56 | 0.15 (0.12,0.19) |
| KIR3DS1*049Ni | 16 | 0.02 (0.01,0.03) | 12 | 0.02 (0.01,0.04) | 4 | 0.01 (0.00,0.03) |

B: Allelic frequencies for all KIR genes using the GAD cohort: Subjects without AD

|  | GAD only without AD | | White no AD | | Black no AD | |
| --- | --- | --- | --- | --- | --- | --- |
| Allele name | n | AF(95% CI) | n | AF(95% CI) | n | AF(95% CI) |
| KIR2DL1*0040101i | 48 | 0.14 (0.10,0.18) | 33 | 0.14 (0.10,0.20) | 13 | 0.11 (0.06,0.18) |
| KIR2DL1*0010101i | 94 | 0.27 (0.22,0.32) | 82 | 0.36 (0.29,0.42) | 12 | 0.10 (0.05,0.17) |
| KIR2DL1*00101i | 2 | 0.01 (0.00,0.02) | 2 | 0.01 (0.00,0.03) | 0 | 0.00 (0.00,0.03) |
| KIR2DL1*00301i | 150 | 0.43 (0.37,0.48) | 84 | 0.37 (0.30,0.43) | 64 | 0.54 (0.45,0.63) |
| KIR2DL1*00303i | 15 | 0.04 (0.02,0.07) | 7 | 0.03 (0.01,0.06) | 8 | 0.07 (0.03,0.13) |
| KIR2DL1*00601i | 5 | 0.01 (0.00,0.03) | 0 | 0.00 (0.00,0.02) | 5 | 0.04 (0.01,0.10) |
| KIR2DL1*007i | 6 | 0.02 (0.01,0.04) | 2 | 0.01 (0.00,0.03) | 4 | 0.03 (0.01,0.08) |
| KIR2DL1*008i | 3 | 0.01 (0.00,0.02) | 3 | 0.01 (0.00,0.04) | 0 | 0.00 (0.00,0.03) |
| KIR2DL1*01201i | 10 | 0.03 (0.01,0.05) | 1 | 0.00 (0.00,0.02) | 9 | 0.08 (0.04,0.14) |
| KIR2DL1*014i | 1 | 0.00 (0.00,0.02) | 0 | 0.00 (0.00,0.02) | 1 | 0.01 (0.00,0.05) |
| KIR2DL1* gene present | 2 | 0.01 (0.00,0.02) | 0 | 0.00 (0.00,0.02) | 2 | 0.02 (0.00,0.06) |
| KIR2DL2*0010101i | 103 | 0.29 (0.25,0.34) | 72 | 0.31 (0.25,0.38) | 30 | 0.25 (0.18,0.34) |
| KIR2DL2*0030101i | 76 | 0.22 (0.17,0.26) | 60 | 0.26 (0.21,0.32) | 15 | 0.13 (0.07,0.20) |
| KIR2DL2*004i | 1 | 0.00 (0.00,0.02) | 0 | 0.00 (0.00,0.02) | 1 | 0.01 (0.00,0.05) |
| KIR2DL2*00601i | 2 | 0.01 (0.00,0.02) | 0 | 0.00 (0.00,0.02) | 2 | 0.02 (0.00,0.06) |
| KIR2DL2*00602i | 2 | 0.01 (0.00,0.02) | 0 | 0.00 (0.00,0.02) | 2 | 0.02 (0.00,0.06) |
| KIR2DL3*0010101i | 265 | 0.75 (0.70,0.80) | 180 | 0.78 (0.72,0.83) | 83 | 0.70 (0.61,0.78) |
| KIR2DL3*00103i | 9 | 0.03 (0.01,0.05) | 4 | 0.02 (0.00,0.04) | 5 | 0.04 (0.01,0.10) |
| KIR2DL3*00501i | 31 | 0.09 (0.06,0.12) | 14 | 0.06 (0.03,0.10) | 17 | 0.14 (0.09,0.22) |
| KIR2DL3*00502i | 2 | 0.01 (0.00,0.02) | 0 | 0.00 (0.00,0.02) | 2 | 0.02 (0.00,0.06) |
| KIR2DL3*011i | 1 | 0.00 (0.00,0.02) | 0 | 0.00 (0.00,0.02) | 1 | 0.01 (0.00,0.05) |
| KIR2DL3*013i | 2 | 0.01 (0.00,0.02) | 0 | 0.00 (0.00,0.02) | 2 | 0.02 (0.00,0.06) |
| KIR2DL3*014 | 2 | 0.01 (0.00,0.02) | 0 | 0.00 (0.00,0.02) | 2 | 0.02 (0.00,0.06) |
| KIR2DL3*014i | 1 | 0.00 (0.00,0.02) | 0 | 0.00 (0.00,0.02) | 1 | 0.01 (0.00,0.05) |
| KIR2DL3*033i | 13 | 0.04 (0.02,0.06) | 12 | 0.05 (0.03,0.09) | 1 | 0.01 (0.00,0.05) |
| KIR2DL4*0010201i | 128 | 0.36 (0.31,0.42) | 74 | 0.32 (0.26,0.39) | 52 | 0.44 (0.35,0.54) |
| KIR2DL4*00201i | 43 | 0.12 (0.09,0.16) | 32 | 0.14 (0.10,0.19) | 11 | 0.09 (0.05,0.16) |
| KIR2DL4*00202i | 63 | 0.18 (0.14,0.22) | 39 | 0.17 (0.12,0.22) | 24 | 0.20 (0.13,0.29) |
| KIR2DL4*0050101i | 97 | 0.28 (0.23,0.33) | 75 | 0.33 (0.27,0.39) | 20 | 0.17 (0.11,0.25) |
| KIR2DL4*00601i | 12 | 0.03 (0.02,0.06) | 8 | 0.03 (0.02,0.07) | 4 | 0.03 (0.01,0.08) |
| KIR2DL4*010i | 3 | 0.01 (0.00,0.02) | 0 | 0.00 (0.00,0.02) | 3 | 0.03 (0.01,0.07) |
| KIR2DL4*018i | 1 | 0.00 (0.00,0.02) | 0 | 0.00 (0.00,0.02) | 1 | 0.01 (0.00,0.05) |
| KIR2DL4*023i | 3 | 0.01 (0.00,0.02) | 0 | 0.00 (0.00,0.02) | 3 | 0.03 (0.01,0.07) |
| KIR2DL5*0020101i | 82 | 0.23 (0.19,0.28) | 58 | 0.25 (0.20,0.31) | 23 | 0.19 (0.13,0.28) |
| KIR2DL5*0010101i | 72 | 0.20 (0.16,0.25) | 48 | 0.21 (0.16,0.27) | 23 | 0.19 (0.13,0.28) |
| KIR2DL5*004i | 4 | 0.01 (0.00,0.03) | 0 | 0.00 (0.00,0.02) | 4 | 0.03 (0.01,0.08) |
| KIR2DP1*0010201i | 47 | 0.13 (0.10,0.17) | 35 | 0.15 (0.11,0.21) | 10 | 0.08 (0.04,0.15) |
| KIR2DP1*00101i | 5 | 0.01 (0.00,0.03) | 4 | 0.02 (0.00,0.04) | 1 | 0.01 (0.00,0.05) |
| KIR2DP1*00103i | 3 | 0.01 (0.00,0.02) | 1 | 0.00 (0.00,0.02) | 2 | 0.02 (0.00,0.06) |
| KIR2DP1*0020101i | 140 | 0.40 (0.35,0.45) | 73 | 0.32 (0.26,0.38) | 65 | 0.55 (0.46,0.64) |
| KIR2DP1*0030101i | 98 | 0.28 (0.23,0.33) | 83 | 0.36 (0.30,0.43) | 15 | 0.13 (0.07,0.20) |
| KIR2DP1*004i | 5 | 0.01 (0.00,0.03) | 5 | 0.02 (0.01,0.05) | 0 | 0.00 (0.00,0.03) |
| KIR2DP1*007i | 9 | 0.03 (0.01,0.05) | 4 | 0.02 (0.00,0.04) | 5 | 0.04 (0.01,0.10) |
| KIR2DP1*008i | 1 | 0.00 (0.00,0.02) | 1 | 0.00 (0.00,0.02) | 0 | 0.00 (0.00,0.03) |
| KIR2DP1*009i | 4 | 0.01 (0.00,0.03) | 0 | 0.00 (0.00,0.02) | 4 | 0.03 (0.01,0.08) |
| KIR2DP1*010i | 7 | 0.02 (0.01,0.04) | 0 | 0.00 (0.00,0.02) | 7 | 0.06 (0.02,0.12) |
| KIR2DP1*013i | 9 | 0.03 (0.01,0.05) | 0 | 0.00 (0.00,0.02) | 9 | 0.08 (0.04,0.14) |
| KIR2DP1*017i | 2 | 0.01 (0.00,0.02) | 2 | 0.01 (0.00,0.03) | 0 | 0.00 (0.00,0.03) |
| KIR2DP1**gene present | 2 | 0.01 (0.00,0.02) | 2 | 0.01 (0.00,0.03) | 0 | 0.00 (0.00,0.03) |
| KIR2DS1*0020101i | 99 | 0.28 (0.23,0.33) | 74 | 0.32 (0.26,0.39) | 23 | 0.19 (0.13,0.28) |
| KIR2DS1*004i | 1 | 0.00 (0.00,0.02) | 0 | 0.00 (0.00,0.02) | 1 | 0.01 (0.00,0.05) |
| KIR2DS2*0010101i | 178 | 0.51 (0.45,0.56) | 132 | 0.57 (0.51,0.64) | 44 | 0.37 (0.29,0.47) |
| KIR2DS2*00104i | 2 | 0.01 (0.00,0.02) | 0 | 0.00 (0.00,0.02) | 2 | 0.02 (0.00,0.06) |
| KIR2DS2*gene present | 2 | 0.01 (0.00,0.02) | 0 | 0.00 (0.00,0.02) | 2 | 0.02 (0.00,0.06) |
| KIR2DS3*00101i | 92 | 0.26 (0.22,0.31) | 64 | 0.28 (0.22,0.34) | 26 | 0.22 (0.15,0.31) |
| KIR2DS3*008i | 2 | 0.01 (0.00,0.02) | 2 | 0.01 (0.00,0.03) | 0 | 0.00 (0.00,0.03) |
| KIR2DS3*015i | 2 | 0.01 (0.00,0.02) | 2 | 0.01 (0.00,0.03) | 0 | 0.00 (0.00,0.03) |
| KIR2DS4*0010101i | 124 | 0.35 (0.30,0.40) | 69 | 0.30 (0.24,0.36) | 53 | 0.45 (0.36,0.54) |
| KIR2DS4*0030101i | 88 | 0.25 (0.21,0.30) | 66 | 0.29 (0.23,0.35) | 22 | 0.19 (0.12,0.27) |
| KIR2DS4*0040101i | 19 | 0.05 (0.03,0.08) | 11 | 0.05 (0.02,0.08) | 8 | 0.07 (0.03,0.13) |
| KIR2DS4*0060101i | 57 | 0.16 (0.13,0.20) | 34 | 0.15 (0.10,0.20) | 23 | 0.19 (0.13,0.28) |
| KIR2DS4*009i | 4 | 0.01 (0.00,0.03) | 0 | 0.00 (0.00,0.02) | 4 | 0.03 (0.01,0.08) |
| KIR2DS4*010i | 31 | 0.09 (0.06,0.12) | 28 | 0.12 (0.08,0.17) | 3 | 0.03 (0.01,0.07) |
| KIR2DS4*010i or 0030101i 016i | 14 | 0.04 (0.02,0.07) | 12 | 0.05 (0.03,0.09) | 2 | 0.02 (0.00,0.06) |
| KIR2DS4*012i | 1 | 0.00 (0.00,0.02) | 0 | 0.00 (0.00,0.02) | 1 | 0.01 (0.00,0.05) |
| KIR2DS5*0020101i | 77 | 0.22 (0.18,0.27) | 58 | 0.25 (0.20,0.31) | 17 | 0.14 (0.09,0.22) |
| KIR2DS5*003i | 1 | 0.00 (0.00,0.02) | 0 | 0.00 (0.00,0.02) | 1 | 0.01 (0.00,0.05) |
| KIR2DS5*00502i | 2 | 0.01 (0.00,0.02) | 0 | 0.00 (0.00,0.02) | 2 | 0.02 (0.00,0.06) |
| KIR2DS5*0060101i | 4 | 0.01 (0.00,0.03) | 0 | 0.00 (0.00,0.02) | 4 | 0.03 (0.01,0.08) |
| KIR2DS5*009i | 4 | 0.01 (0.00,0.03) | 2 | 0.01 (0.00,0.03) | 2 | 0.02 (0.00,0.06) |
| KIR2DS5*011i | 2 | 0.01 (0.00,0.02) | 0 | 0.00 (0.00,0.02) | 2 | 0.02 (0.00,0.06) |
| KIR2DS5*gene present | 2 | 0.01 (0.00,0.02) | 0 | 0.00 (0.00,0.02) | 2 | 0.02 (0.00,0.06) |
| KIR3DL1*0150201i | 47 | 0.13 (0.10,0.17) | 22 | 0.10 (0.06,0.14) | 23 | 0.19 (0.13,0.28) |
| KIR3DL1*0200101i | 14 | 0.04 (0.02,0.07) | 6 | 0.03 (0.01,0.06) | 8 | 0.07 (0.03,0.13) |
| KIR3DL1*0010101i | 61 | 0.17 (0.14,0.22) | 44 | 0.19 (0.14,0.25) | 17 | 0.14 (0.09,0.22) |
| KIR3DL1*0020101i | 41 | 0.12 (0.08,0.15) | 39 | 0.17 (0.12,0.22) | 2 | 0.02 (0.00,0.06) |
| KIR3DL1*0040101i | 46 | 0.13 (0.10,0.17) | 32 | 0.14 (0.10,0.19) | 14 | 0.12 (0.07,0.19) |
| KIR3DL1*0050101i | 44 | 0.13 (0.09,0.16) | 38 | 0.17 (0.12,0.22) | 6 | 0.05 (0.02,0.11) |
| KIR3DL1*0070101i | 41 | 0.12 (0.08,0.15) | 14 | 0.06 (0.03,0.10) | 27 | 0.23 (0.16,0.32) |
| KIR3DL1*0080101i | 18 | 0.05 (0.03,0.08) | 17 | 0.07 (0.04,0.12) | 1 | 0.01 (0.00,0.05) |
| KIR3DL1*0090101i | 7 | 0.02 (0.01,0.04) | 4 | 0.02 (0.00,0.04) | 3 | 0.03 (0.01,0.07) |
| KIR3DL1*019i | 2 | 0.01 (0.00,0.02) | 2 | 0.01 (0.00,0.03) | 0 | 0.00 (0.00,0.03) |
| KIR3DL1*022i | 4 | 0.01 (0.00,0.03) | 0 | 0.00 (0.00,0.02) | 4 | 0.03 (0.01,0.08) |
| KIR3DL1*028i | 3 | 0.01 (0.00,0.02) | 0 | 0.00 (0.00,0.02) | 3 | 0.03 (0.01,0.07) |
| KIR3DL1*033i | 3 | 0.01 (0.00,0.02) | 0 | 0.00 (0.00,0.02) | 3 | 0.03 (0.01,0.07) |
| KIR3DL1*053i | 2 | 0.01 (0.00,0.02) | 2 | 0.01 (0.00,0.03) | 0 | 0.00 (0.00,0.03) |
| KIR3DL1*05901i | 3 | 0.01 (0.00,0.02) | 0 | 0.00 (0.00,0.02) | 3 | 0.03 (0.01,0.07) |
| KIR3DL1*061i | 1 | 0.00 (0.00,0.02) | 0 | 0.00 (0.00,0.02) | 1 | 0.01 (0.00,0.05) |
| KIR3DL1*065i | 1 | 0.00 (0.00,0.02) | 0 | 0.00 (0.00,0.02) | 1 | 0.01 (0.00,0.05) |
| KIR3DL2*0010101i | 136 | 0.39 (0.34,0.44) | 89 | 0.39 (0.32,0.45) | 47 | 0.40 (0.31,0.49) |
| KIR3DL2*0020101i | 41 | 0.12 (0.08,0.15) | 31 | 0.13 (0.09,0.19) | 8 | 0.07 (0.03,0.13) |
| KIR3DL2*00102i | 1 | 0.00 (0.00,0.02) | 1 | 0.00 (0.00,0.02) | 0 | 0.00 (0.00,0.03) |
| KIR3DL2*00301i | 23 | 0.07 (0.04,0.10) | 13 | 0.06 (0.03,0.09) | 10 | 0.08 (0.04,0.15) |
| KIR3DL2*00302i | 6 | 0.02 (0.01,0.04) | 0 | 0.00 (0.00,0.02) | 6 | 0.05 (0.02,0.11) |
| KIR3DL2*00501i | 13 | 0.04 (0.02,0.06) | 12 | 0.05 (0.03,0.09) | 1 | 0.01 (0.00,0.05) |
| KIR3DL2*00501i or 00301i 023i | 5 | 0.01 (0.00,0.03) | 3 | 0.01 (0.00,0.04) | 2 | 0.02 (0.00,0.06) |
| KIR3DL2*00601i | 36 | 0.10 (0.07,0.14) | 24 | 0.10 (0.07,0.15) | 10 | 0.08 (0.04,0.15) |
| KIR3DL2*008i | 6 | 0.02 (0.01,0.04) | 3 | 0.01 (0.00,0.04) | 3 | 0.03 (0.01,0.07) |
| KIR3DL2*008i or 016i 021i | 6 | 0.02 (0.01,0.04) | 4 | 0.02 (0.00,0.04) | 2 | 0.02 (0.00,0.06) |
| KIR3DL2*008i or 016i 027i | 2 | 0.01 (0.00,0.02) | 1 | 0.00 (0.00,0.02) | 1 | 0.01 (0.00,0.05) |
| KIR3DL2*00901i | 15 | 0.04 (0.02,0.07) | 14 | 0.06 (0.03,0.10) | 1 | 0.01 (0.00,0.05) |
| KIR3DL2*00901i or 021i 036i | 1 | 0.00 (0.00,0.02) | 1 | 0.00 (0.00,0.02) | 0 | 0.00 (0.00,0.03) |
| KIR3DL2*00901i or 016i 023i | 3 | 0.01 (0.00,0.02) | 2 | 0.01 (0.00,0.03) | 1 | 0.01 (0.00,0.05) |
| KIR3DL2*01301i | 13 | 0.04 (0.02,0.06) | 0 | 0.00 (0.00,0.02) | 13 | 0.11 (0.06,0.18) |
| KIR3DL2*015i or 0020101i 00601i | 9 | 0.03 (0.01,0.05) | 9 | 0.04 (0.02,0.07) | 0 | 0.00 (0.00,0.03) |
| KIR3DL2*018i | 3 | 0.01 (0.00,0.02) | 3 | 0.01 (0.00,0.04) | 0 | 0.00 (0.00,0.03) |
| KIR3DL2*01901i | 1 | 0.00 (0.00,0.02) | 1 | 0.00 (0.00,0.02) | 0 | 0.00 (0.00,0.03) |
| KIR3DL2*01901i or 00102i 112i or 00601i 00901i | 3 | 0.01 (0.00,0.02) | 1 | 0.00 (0.00,0.02) | 2 | 0.02 (0.00,0.06) |
| KIR3DL2*01901i or 027i 036i | 1 | 0.00 (0.00,0.02) | 1 | 0.00 (0.00,0.02) | 0 | 0.00 (0.00,0.03) |
| KIR3DL2*023i | 3 | 0.01 (0.00,0.02) | 0 | 0.00 (0.00,0.02) | 3 | 0.03 (0.01,0.07) |
| KIR3DL2*025i or 00501i 01301i | 1 | 0.00 (0.00,0.02) | 1 | 0.00 (0.00,0.02) | 0 | 0.00 (0.00,0.03) |
| KIR3DL2*026i or 002010 | 11 | 0.03 (0.02,0.06) | 11 | 0.05 (0.02,0.08) | 0 | 0.00 (0.00,0.03) |
| KIR3DL2*032i | 1 | 0.00 (0.00,0.02) | 0 | 0.00 (0.00,0.02) | 1 | 0.01 (0.00,0.05) |
| KIR3DL2*034i | 1 | 0.00 (0.00,0.02) | 1 | 0.00 (0.00,0.02) | 0 | 0.00 (0.00,0.03) |
| KIR3DL2*037i or 01301i | 1 | 0.00 (0.00,0.02) | 1 | 0.00 (0.00,0.02) | 0 | 0.00 (0.00,0.03) |
| KIR3DL2*039i | 1 | 0.00 (0.00,0.02) | 0 | 0.00 (0.00,0.02) | 1 | 0.01 (0.00,0.05) |
| KIR3DL2*040i | 1 | 0.00 (0.00,0.02) | 0 | 0.00 (0.00,0.02) | 1 | 0.01 (0.00,0.05) |
| KIR3DL2*04301i or 0050 | 1 | 0.00 (0.00,0.02) | 1 | 0.00 (0.00,0.02) | 0 | 0.00 (0.00,0.03) |
| KIR3DL2*04301i or 0060 | 1 | 0.00 (0.00,0.02) | 0 | 0.00 (0.00,0.02) | 1 | 0.01 (0.00,0.05) |
| KIR3DL2*04301i or 015i | 1 | 0.00 (0.00,0.02) | 1 | 0.00 (0.00,0.02) | 0 | 0.00 (0.00,0.03) |
| KIR3DL2*051i | 2 | 0.01 (0.00,0.02) | 0 | 0.00 (0.00,0.02) | 2 | 0.02 (0.00,0.06) |
| KIR3DL2*06201i | 1 | 0.00 (0.00,0.02) | 1 | 0.00 (0.00,0.02) | 0 | 0.00 (0.00,0.03) |
| KIR3DL2*06201i or 0030 | 1 | 0.00 (0.00,0.02) | 0 | 0.00 (0.00,0.02) | 1 | 0.01 (0.00,0.05) |
| KIR3DL2*103i or 002010 | 1 | 0.00 (0.00,0.02) | 0 | 0.00 (0.00,0.02) | 1 | 0.01 (0.00,0.05) |
| KIR3DL3*0020201i | 37 | 0.11 (0.08,0.14) | 27 | 0.12 (0.08,0.17) | 9 | 0.08 (0.04,0.14) |
| KIR3DL3*0040201i | 10 | 0.03 (0.01,0.05) | 2 | 0.01 (0.00,0.03) | 7 | 0.06 (0.02,0.12) |
| KIR3DL3*0010101i | 150 | 0.43 (0.37,0.48) | 111 | 0.48 (0.42,0.55) | 37 | 0.31 (0.23,0.41) |
| KIR3DL3*00103i | 3 | 0.01 (0.00,0.02) | 3 | 0.01 (0.00,0.04) | 0 | 0.00 (0.00,0.03) |
| KIR3DL3*0020101i | 8 | 0.02 (0.01,0.04) | 7 | 0.03 (0.01,0.06) | 1 | 0.01 (0.00,0.05) |
| KIR3DL3*0020201i or 00211i 01308i | 2 | 0.01 (0.00,0.02) | 2 | 0.01 (0.00,0.03) | 0 | 0.00 (0.00,0.03) |
| KIR3DL3*00204i | 5 | 0.01 (0.00,0.03) | 0 | 0.00 (0.00,0.02) | 5 | 0.04 (0.01,0.10) |
| KIR3DL3*0020601i | 5 | 0.01 (0.00,0.03) | 5 | 0.02 (0.01,0.05) | 0 | 0.00 (0.00,0.03) |
| KIR3DL3*0020601i or 00903i 01308i | 6 | 0.02 (0.01,0.04) | 4 | 0.02 (0.00,0.04) | 2 | 0.02 (0.00,0.06) |
| KIR3DL3*0020701i | 14 | 0.04 (0.02,0.07) | 7 | 0.03 (0.01,0.06) | 7 | 0.06 (0.02,0.12) |
| KIR3DL3*00208i | 2 | 0.01 (0.00,0.02) | 0 | 0.00 (0.00,0.02) | 2 | 0.02 (0.00,0.06) |
| KIR3DL3*00210i | 1 | 0.00 (0.00,0.02) | 1 | 0.00 (0.00,0.02) | 0 | 0.00 (0.00,0.03) |
| KIR3DL3*0040201i or 0020101i 066i | 8 | 0.02 (0.01,0.04) | 4 | 0.02 (0.00,0.04) | 4 | 0.03 (0.01,0.08) |
| KIR3DL3*005i | 19 | 0.05 (0.03,0.08) | 1 | 0.00 (0.00,0.02) | 18 | 0.15 (0.09,0.23) |
| KIR3DL3*0060101i | 12 | 0.03 (0.02,0.06) | 10 | 0.04 (0.02,0.08) | 2 | 0.02 (0.00,0.06) |
| KIR3DL3*0060101i or 02102i 023i | 8 | 0.02 (0.01,0.04) | 8 | 0.03 (0.02,0.07) | 0 | 0.00 (0.00,0.03) |
| KIR3DL3*0070101i | 11 | 0.03 (0.02,0.06) | 9 | 0.04 (0.02,0.07) | 2 | 0.02 (0.00,0.06) |
| KIR3DL3*00905i | 2 | 0.01 (0.00,0.02) | 0 | 0.00 (0.00,0.02) | 2 | 0.02 (0.00,0.06) |
| KIR3DL3*01001i | 15 | 0.04 (0.02,0.07) | 10 | 0.04 (0.02,0.08) | 5 | 0.04 (0.01,0.10) |
| KIR3DL3*01101i | 4 | 0.01 (0.00,0.03) | 4 | 0.02 (0.00,0.04) | 0 | 0.00 (0.00,0.03) |
| KIR3DL3*01101i or 012i 02102i | 4 | 0.01 (0.00,0.03) | 4 | 0.02 (0.00,0.04) | 0 | 0.00 (0.00,0.03) |
| KIR3DL3*012i | 7 | 0.02 (0.01,0.04) | 2 | 0.01 (0.00,0.03) | 5 | 0.04 (0.01,0.10) |
| KIR3DL3*012i or 1101i 023i | 2 | 0.01 (0.00,0.02) | 0 | 0.00 (0.00,0.02) | 2 | 0.02 (0.00,0.06) |
| KIR3DL3*01308i | 1 | 0.00 (0.00,0.02) | 1 | 0.00 (0.00,0.02) | 0 | 0.00 (0.00,0.03) |
| KIR3DL3*01502i | 1 | 0.00 (0.00,0.02) | 0 | 0.00 (0.00,0.02) | 1 | 0.01 (0.00,0.05) |
| KIR3DL3*01502i or 01801i 023i | 1 | 0.00 (0.00,0.02) | 0 | 0.00 (0.00,0.02) | 1 | 0.01 (0.00,0.05) |
| KIR3DL3*01801i | 2 | 0.01 (0.00,0.02) | 2 | 0.01 (0.00,0.03) | 0 | 0.00 (0.00,0.03) |
| KIR3DL3*032i | 1 | 0.00 (0.00,0.02) | 0 | 0.00 (0.00,0.02) | 1 | 0.01 (0.00,0.05) |
| KIR3DL3*040i | 3 | 0.01 (0.00,0.02) | 0 | 0.00 (0.00,0.02) | 3 | 0.03 (0.01,0.07) |
| KIR3DL3*044i | 1 | 0.00 (0.00,0.02) | 1 | 0.00 (0.00,0.02) | 0 | 0.00 (0.00,0.03) |
| KIR3DL3*052i | 1 | 0.00 (0.00,0.02) | 0 | 0.00 (0.00,0.02) | 1 | 0.01 (0.00,0.05) |
| KIR3DL3*057i or 066i 069i | 1 | 0.00 (0.00,0.02) | 0 | 0.00 (0.00,0.02) | 1 | 0.01 (0.00,0.05) |
| KIR3DL3*074i | 2 | 0.01 (0.00,0.02) | 2 | 0.01 (0.00,0.03) | 0 | 0.00 (0.00,0.03) |
| KIR3DL3*10701i or 00103i 102i | 1 | 0.00 (0.00,0.02) | 1 | 0.00 (0.00,0.02) | 0 | 0.00 (0.00,0.03) |
| KIR3DP1*0030101i | 57 | 0.16 (0.13,0.20) | 36 | 0.16 (0.11,0.21) | 19 | 0.16 (0.10,0.24) |
| KIR3DP1*001i | 196 | 0.56 (0.50,0.61) | 110 | 0.48 (0.41,0.54) | 84 | 0.71 (0.62,0.79) |
| KIR3DP1*005i | 83 | 0.24 (0.19,0.28) | 71 | 0.31 (0.25,0.37) | 12 | 0.10 (0.05,0.17) |
| KIR3DP1*007i | 1 | 0.00 (0.00,0.02) | 0 | 0.00 (0.00,0.02) | 1 | 0.01 (0.00,0.05) |
| KIR3DP1*008i | 2 | 0.01 (0.00,0.02) | 0 | 0.00 (0.00,0.02) | 2 | 0.02 (0.00,0.06) |
| KIR3DP1*0140101i | 9 | 0.03 (0.01,0.05) | 9 | 0.04 (0.02,0.07) | 0 | 0.00 (0.00,0.03) |
| KIR3DS1*010i | 88 | 0.25 (0.21,0.30) | 64 | 0.28 (0.22,0.34) | 22 | 0.19 (0.12,0.27) |
| KIR3DS1*049Ni | 4 | 0.01 (0.00,0.03) | 4 | 0.02 (0.00,0.04) | 0 | 0.00 (0.00,0.03) |

C: Allelic frequencies for all KIR genes: GAD cohort subjects with AD

|  | GAD AD all | | White AD | | Black AD | |
| --- | --- | --- | --- | --- | --- | --- |
| Allele name | n | AF(95% CI) | n | AF(95% CI) | n | AF(95% CI) |
| KIR2DL1*0040101i | 91 | 0.15 (0.12,0.18) | 43 | 0.13 (0.10,0.17) | 44 | 0.18 (0.13,0.23) |
| KIR2DL1*0010101i | 149 | 0.24 (0.21,0.27) | 112 | 0.34 (0.29,0.39) | 28 | 0.11 (0.08,0.16) |
| KIR2DL1*00301i | 287 | 0.46 (0.42,0.50) | 146 | 0.44 (0.39,0.49) | 113 | 0.45 (0.39,0.52) |
| KIR2DL1*00303i | 18 | 0.03 (0.02,0.05) | 7 | 0.02 (0.01,0.04) | 12 | 0.05 (0.03,0.08) |
| KIR2DL1*00404i | 5 | 0.01 (0.00,0.02) | 2 | 0.01 (0.00,0.02) | 3 | 0.01 (0.00,0.03) |
| KIR2DL1*00601i | 8 | 0.01 (0.01,0.03) | 1 | 0.00 (0.00,0.02) | 7 | 0.03 (0.01,0.06) |
| KIR2DL1*007i | 18 | 0.03 (0.02,0.05) | 6 | 0.02 (0.01,0.04) | 10 | 0.04 (0.02,0.07) |
| KIR2DL1*008i | 1 | 0.00 (0.00,0.01) | 1 | 0.00 (0.00,0.02) | 0 | 0.00 (0.00,0.01) |
| KIR2DL1*01201i | 18 | 0.03 (0.02,0.05) | 6 | 0.02 (0.01,0.04) | 12 | 0.05 (0.03,0.08) |
| KIR2DL1*013Ni | 1 | 0.00 (0.00,0.01) | 0 | 0.00 (0.00,0.01) | 1 | 0.00 (0.00,0.02) |
| KIR2DL1*014i | 3 | 0.00 (0.00,0.01) | 0 | 0.00 (0.00,0.01) | 3 | 0.01 (0.00,0.03) |
| KIR2DL1*029i | 1 | 0.00 (0.00,0.01) | 0 | 0.00 (0.00,0.01) | 1 | 0.00 (0.00,0.02) |
| KIR2DL1*0320101Ni | 4 | 0.01 (0.00,0.02) | 4 | 0.01 (0.00,0.03) | 0 | 0.00 (0.00,0.01) |
| KIR2DL1**gene present | 6 | 0.01 (0.00,0.02) | 0 | 0.00 (0.00,0.01) | 6 | 0.02 (0.01,0.05) |
| KIR2DL2*0010101i | 176 | 0.28 (0.25,0.32) | 96 | 0.29 (0.24,0.34) | 69 | 0.28 (0.22,0.34) |
| KIR2DL2*00102i | 3 | 0.00 (0.00,0.01) | 0 | 0.00 (0.00,0.01) | 3 | 0.01 (0.00,0.03) |
| KIR2DL2*0030101i | 122 | 0.19 (0.16,0.23) | 75 | 0.23 (0.18,0.27) | 40 | 0.16 (0.12,0.21) |
| KIR2DL2*004i | 6 | 0.01 (0.00,0.02) | 0 | 0.00 (0.00,0.01) | 6 | 0.02 (0.01,0.05) |
| KIR2DL2*00601i | 2 | 0.00 (0.00,0.01) | 0 | 0.00 (0.00,0.01) | 2 | 0.01 (0.00,0.03) |
| KIR2DL2*00602i | 16 | 0.03 (0.01,0.04) | 0 | 0.00 (0.00,0.01) | 16 | 0.06 (0.04,0.10) |
| KIR2DL2*00602i or 00602i 009i | 1 | 0.00 (0.00,0.01) | 1 | 0.00 (0.00,0.02) | 0 | 0.00 (0.00,0.01) |
| KIR2DL2*009i | 6 | 0.01 (0.00,0.02) | 2 | 0.01 (0.00,0.02) | 4 | 0.02 (0.00,0.04) |
| KIR2DL2*0602i | 4 | 0.01 (0.00,0.02) | 0 | 0.00 (0.00,0.01) | 4 | 0.02 (0.00,0.04) |
| KIR2DL3*0010101i | 452 | 0.72 (0.69,0.76) | 261 | 0.79 (0.74,0.83) | 154 | 0.62 (0.55,0.68) |
| KIR2DL3*00103i | 13 | 0.02 (0.01,0.04) | 3 | 0.01 (0.00,0.03) | 11 | 0.04 (0.02,0.08) |
| KIR2DL3*00110i | 5 | 0.01 (0.00,0.02) | 5 | 0.02 (0.00,0.03) | 0 | 0.00 (0.00,0.01) |
| KIR2DL3*004i | 1 | 0.00 (0.00,0.01) | 0 | 0.00 (0.00,0.01) | 1 | 0.00 (0.00,0.02) |
| KIR2DL3*00501i | 66 | 0.11 (0.08,0.13) | 30 | 0.09 (0.06,0.13) | 34 | 0.14 (0.10,0.18) |
| KIR2DL3*009i | 1 | 0.00 (0.00,0.01) | 1 | 0.00 (0.00,0.02) | 0 | 0.00 (0.00,0.01) |
| KIR2DL3*011i | 3 | 0.00 (0.00,0.01) | 0 | 0.00 (0.00,0.01) | 3 | 0.01 (0.00,0.03) |
| KIR2DL3*013i | 5 | 0.01 (0.00,0.02) | 0 | 0.00 (0.00,0.01) | 5 | 0.02 (0.01,0.05) |
| KIR2DL3*014i | 7 | 0.01 (0.00,0.02) | 0 | 0.00 (0.00,0.01) | 7 | 0.03 (0.01,0.06) |
| KIR2DL3*015i | 1 | 0.00 (0.00,0.01) | 1 | 0.00 (0.00,0.02) | 0 | 0.00 (0.00,0.01) |
| KIR2DL3*033i | 10 | 0.02 (0.01,0.03) | 9 | 0.03 (0.01,0.05) | 1 | 0.00 (0.00,0.02) |
| KIR2DL3*gene present | 6 | 0.01 (0.00,0.02) | 0 | 0.00 (0.00,0.01) | 4 | 0.02 (0.00,0.04) |
| KIR2DL4*0010201i | 226 | 0.36 (0.32,0.40) | 95 | 0.29 (0.24,0.34) | 113 | 0.45 (0.39,0.52) |
| KIR2DL4*00201i | 87 | 0.14 (0.11,0.17) | 54 | 0.16 (0.12,0.21) | 33 | 0.13 (0.09,0.18) |
| KIR2DL4*00202i | 87 | 0.14 (0.11,0.17) | 52 | 0.16 (0.12,0.20) | 27 | 0.11 (0.07,0.15) |
| KIR2DL4*0050101i | 186 | 0.30 (0.26,0.33) | 119 | 0.36 (0.31,0.41) | 53 | 0.21 (0.16,0.27) |
| KIR2DL4*00601i | 30 | 0.05 (0.03,0.07) | 11 | 0.03 (0.02,0.06) | 15 | 0.06 (0.03,0.10) |
| KIR2DL4*010i | 4 | 0.01 (0.00,0.02) | 0 | 0.00 (0.00,0.01) | 4 | 0.02 (0.00,0.04) |
| KIR2DL4*017i | 1 | 0.00 (0.00,0.01) | 0 | 0.00 (0.00,0.01) | 1 | 0.00 (0.00,0.02) |
| KIR2DL4*021i | 2 | 0.00 (0.00,0.01) | 0 | 0.00 (0.00,0.01) | 2 | 0.01 (0.00,0.03) |
| KIR2DL4*023i | 3 | 0.00 (0.00,0.01) | 1 | 0.00 (0.00,0.02) | 2 | 0.01 (0.00,0.03) |
| KIR2DL5*0020101i | 141 | 0.23 (0.19,0.26) | 79 | 0.24 (0.19,0.29) | 48 | 0.19 (0.15,0.25) |
| KIR2DL5*0010101i | 204 | 0.33 (0.29,0.36) | 97 | 0.29 (0.24,0.34) | 92 | 0.37 (0.31,0.43) |
| KIR2DL5*00103i | 1 | 0.00 (0.00,0.01) | 1 | 0.00 (0.00,0.02) | 0 | 0.00 (0.00,0.01) |
| KIR2DL5*00203i | 5 | 0.01 (0.00,0.02) | 1 | 0.00 (0.00,0.02) | 3 | 0.01 (0.00,0.03) |
| KIR2DL5*003i | 4 | 0.01 (0.00,0.02) | 0 | 0.00 (0.00,0.01) | 4 | 0.02 (0.00,0.04) |
| KIR2DL5*004i | 1 | 0.00 (0.00,0.01) | 0 | 0.00 (0.00,0.01) | 1 | 0.00 (0.00,0.02) |
| KIR2DP1*0010201i | 79 | 0.13 (0.10,0.15) | 47 | 0.14 (0.11,0.18) | 26 | 0.10 (0.07,0.15) |
| KIR2DP1*00101i | 8 | 0.01 (0.01,0.03) | 7 | 0.02 (0.01,0.04) | 0 | 0.00 (0.00,0.01) |
| KIR2DP1*00103i | 6 | 0.01 (0.00,0.02) | 0 | 0.00 (0.00,0.01) | 6 | 0.02 (0.01,0.05) |
| KIR2DP1*0020101i | 253 | 0.40 (0.37,0.44) | 126 | 0.38 (0.33,0.43) | 106 | 0.42 (0.36,0.49) |
| KIR2DP1*00204i | 2 | 0.00 (0.00,0.01) | 2 | 0.01 (0.00,0.02) | 0 | 0.00 (0.00,0.01) |
| KIR2DP1*0030101i | 166 | 0.27 (0.23,0.30) | 116 | 0.35 (0.30,0.40) | 36 | 0.14 (0.10,0.19) |
| KIR2DP1*004i | 6 | 0.01 (0.00,0.02) | 5 | 0.02 (0.00,0.03) | 1 | 0.00 (0.00,0.02) |
| KIR2DP1*007i | 10 | 0.02 (0.01,0.03) | 1 | 0.00 (0.00,0.02) | 10 | 0.04 (0.02,0.07) |
| KIR2DP1*008i | 6 | 0.01 (0.00,0.02) | 6 | 0.02 (0.01,0.04) | 0 | 0.00 (0.00,0.01) |
| KIR2DP1*009i | 8 | 0.01 (0.01,0.03) | 0 | 0.00 (0.00,0.01) | 8 | 0.03 (0.01,0.06) |
| KIR2DP1*010i | 28 | 0.04 (0.03,0.06) | 4 | 0.01 (0.00,0.03) | 24 | 0.10 (0.06,0.14) |
| KIR2DP1*013i | 9 | 0.01 (0.01,0.03) | 1 | 0.00 (0.00,0.02) | 8 | 0.03 (0.01,0.06) |
| KIR2DP1*015i | 1 | 0.00 (0.00,0.01) | 0 | 0.00 (0.00,0.01) | 1 | 0.00 (0.00,0.02) |
| KIR2DP1*017i | 5 | 0.01 (0.00,0.02) | 5 | 0.02 (0.00,0.03) | 0 | 0.00 (0.00,0.01) |
| KIR2DP1*019i | 1 | 0.00 (0.00,0.01) | 0 | 0.00 (0.00,0.01) | 0 | 0.00 (0.00,0.01) |
| KIR2DP1*gene present | 16 | 0.03 (0.01,0.04) | 6 | 0.02 (0.01,0.04) | 10 | 0.04 (0.02,0.07) |
| KIR2DS1*0020101i | 207 | 0.33 (0.29,0.37) | 135 | 0.41 (0.35,0.46) | 50 | 0.20 (0.15,0.26) |
| KIR2DS1*004i | 8 | 0.01 (0.01,0.03) | 0 | 0.00 (0.00,0.01) | 8 | 0.03 (0.01,0.06) |
| KIR2DS1*00501i | 5 | 0.01 (0.00,0.02) | 3 | 0.01 (0.00,0.03) | 2 | 0.01 (0.00,0.03) |
| KIR2DS1*gene present | 8 | 0.01 (0.01,0.03) | 2 | 0.01 (0.00,0.02) | 6 | 0.02 (0.01,0.05) |
| KIR2DS2*0010101i | 319 | 0.51 (0.47,0.55) | 174 | 0.52 (0.47,0.58) | 127 | 0.51 (0.44,0.57) |
| KIR2DS2*0010701i | 1 | 0.00 (0.00,0.01) | 0 | 0.00 (0.00,0.01) | 1 | 0.00 (0.00,0.02) |
| KIR2DS2*016i | 2 | 0.00 (0.00,0.01) | 0 | 0.00 (0.00,0.01) | 2 | 0.01 (0.00,0.03) |
| KIR2DS3*00101i | 163 | 0.26 (0.23,0.30) | 95 | 0.29 (0.24,0.34) | 54 | 0.22 (0.17,0.27) |
| KIR2DS3*0030101Ni | 5 | 0.01 (0.00,0.02) | 5 | 0.02 (0.00,0.03) | 0 | 0.00 (0.00,0.01) |
| KIR2DS3*017i | 2 | 0.00 (0.00,0.01) | 0 | 0.00 (0.00,0.01) | 0 | 0.00 (0.00,0.01) |
| KIR2DS3*0210101i | 2 | 0.00 (0.00,0.01) | 0 | 0.00 (0.00,0.01) | 2 | 0.01 (0.00,0.03) |
| KIR2DS3*0220101i | 2 | 0.00 (0.00,0.01) | 2 | 0.01 (0.00,0.02) | 0 | 0.00 (0.00,0.01) |
| KIR2DS4*0010101i | 244 | 0.39 (0.35,0.43) | 101 | 0.30 (0.26,0.36) | 120 | 0.48 (0.42,0.54) |
| KIR2DS4*0030101i | 129 | 0.21 (0.18,0.24) | 85 | 0.26 (0.21,0.31) | 31 | 0.12 (0.09,0.17) |
| KIR2DS4*0040101i | 37 | 0.06 (0.04,0.08) | 12 | 0.04 (0.02,0.06) | 21 | 0.08 (0.05,0.13) |
| KIR2DS4*0060101i | 105 | 0.17 (0.14,0.20) | 66 | 0.20 (0.16,0.25) | 39 | 0.16 (0.11,0.21) |
| KIR2DS4*009i | 8 | 0.01 (0.01,0.03) | 0 | 0.00 (0.00,0.01) | 8 | 0.03 (0.01,0.06) |
| KIR2DS4*010i | 56 | 0.09 (0.07,0.11) | 41 | 0.12 (0.09,0.16) | 15 | 0.06 (0.03,0.10) |
| KIR2DS4*010i or 0030101i 016i | 15 | 0.02 (0.01,0.04) | 9 | 0.03 (0.01,0.05) | 4 | 0.02 (0.00,0.04) |
| KIR2DS4*012i | 2 | 0.00 (0.00,0.01) | 0 | 0.00 (0.00,0.01) | 2 | 0.01 (0.00,0.03) |
| KIR2DS4*013i | 2 | 0.00 (0.00,0.01) | 0 | 0.00 (0.00,0.01) | 2 | 0.01 (0.00,0.03) |
| KIR2DS5*0020101i | 165 | 0.26 (0.23,0.30) | 114 | 0.34 (0.29,0.40) | 37 | 0.15 (0.11,0.20) |
| KIR2DS5*003i | 7 | 0.01 (0.00,0.02) | 0 | 0.00 (0.00,0.01) | 7 | 0.03 (0.01,0.06) |
| KIR2DS5*00501i | 31 | 0.05 (0.03,0.07) | 2 | 0.01 (0.00,0.02) | 29 | 0.12 (0.08,0.16) |
| KIR2DS5*00502i | 1 | 0.00 (0.00,0.01) | 0 | 0.00 (0.00,0.01) | 1 | 0.00 (0.00,0.02) |
| KIR2DS5*0060101i | 5 | 0.01 (0.00,0.02) | 0 | 0.00 (0.00,0.01) | 5 | 0.02 (0.01,0.05) |
| KIR2DS5*009i | 15 | 0.02 (0.01,0.04) | 0 | 0.00 (0.00,0.01) | 15 | 0.06 (0.03,0.10) |
| KIR2DS5*010i | 2 | 0.00 (0.00,0.01) | 0 | 0.00 (0.00,0.01) | 2 | 0.01 (0.00,0.03) |
| KIR2DS5*011i | 2 | 0.00 (0.00,0.01) | 0 | 0.00 (0.00,0.01) | 2 | 0.01 (0.00,0.03) |
| KIR2DS5*gene present | 2 | 0.00 (0.00,0.01) | 0 | 0.00 (0.00,0.01) | 2 | 0.01 (0.00,0.03) |
| KIR3DL1*0150201i | 100 | 0.16 (0.13,0.19) | 48 | 0.14 (0.11,0.19) | 35 | 0.14 (0.10,0.19) |
| KIR3DL1*0200101i | 20 | 0.03 (0.02,0.05) | 8 | 0.02 (0.01,0.05) | 10 | 0.04 (0.02,0.07) |
| KIR3DL1*0010101i | 95 | 0.15 (0.12,0.18) | 61 | 0.18 (0.14,0.23) | 25 | 0.10 (0.07,0.14) |
| KIR3DL1*0020101i | 51 | 0.08 (0.06,0.11) | 35 | 0.11 (0.07,0.14) | 13 | 0.05 (0.03,0.09) |
| KIR3DL1*0040101i | 88 | 0.14 (0.11,0.17) | 59 | 0.18 (0.14,0.22) | 29 | 0.12 (0.08,0.16) |
| KIR3DL1*0050101i | 66 | 0.11 (0.08,0.13) | 46 | 0.14 (0.10,0.18) | 18 | 0.07 (0.04,0.11) |
| KIR3DL1*0070101i | 100 | 0.16 (0.13,0.19) | 20 | 0.06 (0.04,0.09) | 75 | 0.30 (0.24,0.36) |
| KIR3DL1*0080101i | 22 | 0.04 (0.02,0.05) | 17 | 0.05 (0.03,0.08) | 2 | 0.01 (0.00,0.03) |
| KIR3DL1*0090101i | 7 | 0.01 (0.00,0.02) | 7 | 0.02 (0.01,0.04) | 0 | 0.00 (0.00,0.01) |
| KIR3DL1*0090101i or 0070101i 054i | 1 | 0.00 (0.00,0.01) | 0 | 0.00 (0.00,0.01) | 0 | 0.00 (0.00,0.01) |
| KIR3DL1*019i | 6 | 0.01 (0.00,0.02) | 6 | 0.02 (0.01,0.04) | 0 | 0.00 (0.00,0.01) |
| KIR3DL1*022i | 8 | 0.01 (0.01,0.03) | 0 | 0.00 (0.00,0.01) | 8 | 0.03 (0.01,0.06) |
| KIR3DL1*023i | 1 | 0.00 (0.00,0.01) | 0 | 0.00 (0.00,0.01) | 1 | 0.00 (0.00,0.02) |
| KIR3DL1*028i | 2 | 0.00 (0.00,0.01) | 0 | 0.00 (0.00,0.01) | 2 | 0.01 (0.00,0.03) |
| KIR3DL1*0290101i | 2 | 0.00 (0.00,0.01) | 2 | 0.01 (0.00,0.02) | 0 | 0.00 (0.00,0.01) |
| KIR3DL1*033i | 9 | 0.01 (0.01,0.03) | 0 | 0.00 (0.00,0.01) | 9 | 0.04 (0.02,0.07) |
| KIR3DL1*03501i | 1 | 0.00 (0.00,0.01) | 0 | 0.00 (0.00,0.01) | 1 | 0.00 (0.00,0.02) |
| KIR3DL1*042i | 3 | 0.00 (0.00,0.01) | 3 | 0.01 (0.00,0.03) | 0 | 0.00 (0.00,0.01) |
| KIR3DL1*053i | 3 | 0.00 (0.00,0.01) | 1 | 0.00 (0.00,0.02) | 2 | 0.01 (0.00,0.03) |
| KIR3DL1*05901i | 7 | 0.01 (0.00,0.02) | 0 | 0.00 (0.00,0.01) | 7 | 0.03 (0.01,0.06) |
| KIR3DL1*063i | 2 | 0.00 (0.00,0.01) | 0 | 0.00 (0.00,0.01) | 2 | 0.01 (0.00,0.03) |
| KIR3DL1*072i | 1 | 0.00 (0.00,0.01) | 1 | 0.00 (0.00,0.02) | 0 | 0.00 (0.00,0.01) |
| KIR3DL1*089i | 1 | 0.00 (0.00,0.01) | 0 | 0.00 (0.00,0.01) | 1 | 0.00 (0.00,0.02) |
| KIR3DL1*090101i | 1 | 0.00 (0.00,0.01) | 0 | 0.00 (0.00,0.01) | 1 | 0.00 (0.00,0.02) |
| KIR3DL1*092i | 1 | 0.00 (0.00,0.01) | 0 | 0.00 (0.00,0.01) | 1 | 0.00 (0.00,0.02) |
| KIR3DL2*0010101i | 214 | 0.34 (0.30,0.38) | 105 | 0.32 (0.27,0.37) | 92 | 0.37 (0.31,0.43) |
| KIR3DL2*0020101i | 78 | 0.12 (0.10,0.15) | 53 | 0.16 (0.12,0.20) | 17 | 0.07 (0.04,0.11) |
| KIR3DL2*00301i | 48 | 0.08 (0.06,0.10) | 31 | 0.09 (0.06,0.13) | 17 | 0.07 (0.04,0.11) |
| KIR3DL2*00302i | 11 | 0.02 (0.01,0.03) | 0 | 0.00 (0.00,0.01) | 11 | 0.04 (0.02,0.08) |
| KIR3DL2*00501i | 11 | 0.02 (0.01,0.03) | 9 | 0.03 (0.01,0.05) | 2 | 0.01 (0.00,0.03) |
| KIR3DL2*00501i or 00301i 023i | 14 | 0.02 (0.01,0.04) | 14 | 0.04 (0.02,0.07) | 0 | 0.00 (0.00,0.01) |
| KIR3DL2*00501i or 023i 026i | 5 | 0.01 (0.00,0.02) | 5 | 0.02 (0.00,0.03) | 0 | 0.00 (0.00,0.01) |
| KIR3DL2*00601i | 73 | 0.12 (0.09,0.14) | 41 | 0.12 (0.09,0.16) | 29 | 0.12 (0.08,0.16) |
| KIR3DL2*008i | 14 | 0.02 (0.01,0.04) | 5 | 0.02 (0.00,0.03) | 7 | 0.03 (0.01,0.06) |
| KIR3DL2*008i or 016i 021i | 11 | 0.02 (0.01,0.03) | 5 | 0.02 (0.00,0.03) | 4 | 0.02 (0.00,0.04) |
| KIR3DL2*008i or 016i 027i | 5 | 0.01 (0.00,0.02) | 0 | 0.00 (0.00,0.01) | 5 | 0.02 (0.01,0.05) |
| KIR3DL2*0090101i | 1 | 0.00 (0.00,0.01) | 0 | 0.00 (0.00,0.01) | 0 | 0.00 (0.00,0.01) |
| KIR3DL2*00901i | 24 | 0.04 (0.02,0.06) | 13 | 0.04 (0.02,0.07) | 9 | 0.04 (0.02,0.07) |
| KIR3DL2*01901i or 00102i 112i or 00601i 00901i | 1 | 0.00 (0.00,0.01) | 1 | 0.00 (0.00,0.02) | 0 | 0.00 (0.00,0.01) |
| KIR3DL2*012i | 1 | 0.00 (0.00,0.01) | 1 | 0.00 (0.00,0.02) | 0 | 0.00 (0.00,0.01) |
| KIR3DL2*01301i | 28 | 0.04 (0.03,0.06) | 2 | 0.01 (0.00,0.02) | 26 | 0.10 (0.07,0.15) |
| KIR3DL2*015i or 0020101i 00601i | 19 | 0.03 (0.02,0.05) | 13 | 0.04 (0.02,0.07) | 1 | 0.00 (0.00,0.02) |
| KIR3DL2*018i | 8 | 0.01 (0.01,0.03) | 7 | 0.02 (0.01,0.04) | 1 | 0.00 (0.00,0.02) |
| KIR3DL2*01901i | 2 | 0.00 (0.00,0.01) | 0 | 0.00 (0.00,0.01) | 2 | 0.01 (0.00,0.03) |
| KIR3DL2*01901i or 00102i 112i or 00601i 00901i | 7 | 0.01 (0.00,0.02) | 5 | 0.02 (0.00,0.03) | 1 | 0.00 (0.00,0.02) |
| KIR3DL2*01901i or 00601i 06201i | 1 | 0.00 (0.00,0.01) | 1 | 0.00 (0.00,0.02) | 0 | 0.00 (0.00,0.01) |
| KIR3DL2*019 01i or 00901i 015i | 3 | 0.00 (0.00,0.01) | 1 | 0.00 (0.00,0.02) | 2 | 0.01 (0.00,0.03) |
| KIR3DL2*020i | 1 | 0.00 (0.00,0.01) | 0 | 0.00 (0.00,0.01) | 1 | 0.00 (0.00,0.02) |
| KIR3DL2*021i | 1 | 0.00 (0.00,0.01) | 1 | 0.00 (0.00,0.02) | 0 | 0.00 (0.00,0.01) |
| KIR3DL2*023i | 2 | 0.00 (0.00,0.01) | 0 | 0.00 (0.00,0.01) | 2 | 0.01 (0.00,0.03) |
| KIR3DL2*025i or 01301i 023i | 1 | 0.00 (0.00,0.01) | 0 | 0.00 (0.00,0.01) | 1 | 0.00 (0.00,0.02) |
| KIR3DL2*026i or 0020101i 00301i | 6 | 0.01 (0.00,0.02) | 4 | 0.01 (0.00,0.03) | 1 | 0.00 (0.00,0.02) |
| KIR3DL2*027i | 1 | 0.00 (0.00,0.01) | 1 | 0.00 (0.00,0.02) | 0 | 0.00 (0.00,0.01) |
| KIR3DL2*027i or 00601i 021i i | 1 | 0.00 (0.00,0.01) | 1 | 0.00 (0.00,0.02) | 0 | 0.00 (0.00,0.01) |
| KIR3DL2*029i | 6 | 0.01 (0.00,0.02) | 1 | 0.00 (0.00,0.02) | 5 | 0.02 (0.01,0.05) |
| KIR3DL2*033i | 1 | 0.00 (0.00,0.01) | 0 | 0.00 (0.00,0.01) | 1 | 0.00 (0.00,0.02) |
| KIR3DL2*034i | 1 | 0.00 (0.00,0.01) | 1 | 0.00 (0.00,0.02) | 0 | 0.00 (0.00,0.01) |
| KIR3DL2*037i or 01301i | 1 | 0.00 (0.00,0.01) | 0 | 0.00 (0.00,0.01) | 1 | 0.00 (0.00,0.02) |
| KIR3DL2*040i | 3 | 0.00 (0.00,0.01) | 1 | 0.00 (0.00,0.02) | 2 | 0.01 (0.00,0.03) |
| KIR3DL2*04301i or 00500 | 7 | 0.01 (0.00,0.02) | 6 | 0.02 (0.01,0.04) | 1 | 0.00 (0.00,0.02) |
| KIR3DL2*051i | 1 | 0.00 (0.00,0.01) | 1 | 0.00 (0.00,0.02) | 0 | 0.00 (0.00,0.01) |
| KIR3DL2*059i or 023i02 | 1 | 0.00 (0.00,0.01) | 0 | 0.00 (0.00,0.01) | 1 | 0.00 (0.00,0.02) |
| KIR3DL2*06201i | 1 | 0.00 (0.00,0.01) | 0 | 0.00 (0.00,0.01) | 1 | 0.00 (0.00,0.02) |
| KIR3DL2*06201i or 0030 | 1 | 0.00 (0.00,0.01) | 1 | 0.00 (0.00,0.02) | 0 | 0.00 (0.00,0.01) |
| KIR3DL2*064i | 1 | 0.00 (0.00,0.01) | 0 | 0.00 (0.00,0.01) | 0 | 0.00 (0.00,0.01) |
| KIR3DL2*080i | 1 | 0.00 (0.00,0.01) | 0 | 0.00 (0.00,0.01) | 1 | 0.00 (0.00,0.02) |
| KIR3DL2*082i | 1 | 0.00 (0.00,0.01) | 1 | 0.00 (0.00,0.02) | 0 | 0.00 (0.00,0.01) |
| KIR3DL2*083i | 1 | 0.00 (0.00,0.01) | 1 | 0.00 (0.00,0.02) | 0 | 0.00 (0.00,0.01) |
| KIR3DL2*095i | 2 | 0.00 (0.00,0.01) | 0 | 0.00 (0.00,0.01) | 2 | 0.01 (0.00,0.03) |
| KIR3DL2*103i or 0020101I | 2 | 0.00 (0.00,0.01) | 0 | 0.00 (0.00,0.01) | 1 | 0.00 (0.00,0.02) |
| KIR3DL2*1301i or 00201 | 1 | 0.00 (0.00,0.01) | 0 | 0.00 (0.00,0.01) | 1 | 0.00 (0.00,0.02) |
| KIR3DL3*0020201i | 82 | 0.13 (0.11,0.16) | 60 | 0.18 (0.14,0.23) | 19 | 0.08 (0.05,0.12) |
| KIR3DL3*0040201i | 22 | 0.04 (0.02,0.05) | 8 | 0.02 (0.01,0.05) | 11 | 0.04 (0.02,0.08) |
| KIR3DL3*0010101i | 216 | 0.35 (0.31,0.38) | 134 | 0.40 (0.35,0.46) | 67 | 0.27 (0.21,0.33) |
| KIR3DL3*00103i | 8 | 0.01 (0.01,0.03) | 3 | 0.01 (0.00,0.03) | 4 | 0.02 (0.00,0.04) |
| KIR3DL3*00104i | 1 | 0.00 (0.00,0.01) | 1 | 0.00 (0.00,0.02) | 0 | 0.00 (0.00,0.01) |
| KIR3DL3*0020101i | 17 | 0.03 (0.02,0.04) | 12 | 0.04 (0.02,0.06) | 5 | 0.02 (0.01,0.05) |
| KIR3DL3*0020201i or 00211i 01308i | 3 | 0.00 (0.00,0.01) | 3 | 0.01 (0.00,0.03) | 0 | 0.00 (0.00,0.01) |
| KIR3DL3*00204i | 7 | 0.01 (0.00,0.02) | 1 | 0.00 (0.00,0.02) | 6 | 0.02 (0.01,0.05) |
| KIR3DL3*0020601i | 13 | 0.02 (0.01,0.04) | 11 | 0.03 (0.02,0.06) | 2 | 0.01 (0.00,0.03) |
| KIR3DL3*0020601i or 00903i 01308i | 6 | 0.01 (0.00,0.02) | 4 | 0.01 (0.00,0.03) | 2 | 0.01 (0.00,0.03) |
| KIR3DL3*0020701i | 14 | 0.02 (0.01,0.04) | 5 | 0.02 (0.00,0.03) | 8 | 0.03 (0.01,0.06) |
| KIR3DL3*00208i | 18 | 0.03 (0.02,0.05) | 2 | 0.01 (0.00,0.02) | 15 | 0.06 (0.03,0.10) |
| KIR3DL3*00210i | 1 | 0.00 (0.00,0.01) | 0 | 0.00 (0.00,0.01) | 0 | 0.00 (0.00,0.01) |
| KIR3DL3*00211i | 2 | 0.00 (0.00,0.01) | 0 | 0.00 (0.00,0.01) | 2 | 0.01 (0.00,0.03) |
| KIR3DL3*0040201i or 0020101i 066i | 8 | 0.01 (0.01,0.03) | 3 | 0.01 (0.00,0.03) | 4 | 0.02 (0.00,0.04) |
| KIR3DL3*005i | 35 | 0.06 (0.04,0.08) | 1 | 0.00 (0.00,0.02) | 33 | 0.13 (0.09,0.18) |
| KIR3DL3*0060101i | 19 | 0.03 (0.02,0.05) | 14 | 0.04 (0.02,0.07) | 2 | 0.01 (0.00,0.03) |
| KIR3DL3*0060101i or 02102i 023i | 9 | 0.01 (0.01,0.03) | 8 | 0.02 (0.01,0.05) | 1 | 0.00 (0.00,0.02) |
| KIR3DL3*0070101i | 18 | 0.03 (0.02,0.05) | 13 | 0.04 (0.02,0.07) | 3 | 0.01 (0.00,0.03) |
| KIR3DL3*00903i | 2 | 0.00 (0.00,0.01) | 0 | 0.00 (0.00,0.01) | 2 | 0.01 (0.00,0.03) |
| KIR3DL3*00905i | 1 | 0.00 (0.00,0.01) | 0 | 0.00 (0.00,0.01) | 1 | 0.00 (0.00,0.02) |
| KIR3DL3*01001i | 31 | 0.05 (0.03,0.07) | 14 | 0.04 (0.02,0.07) | 9 | 0.04 (0.02,0.07) |
| KIR3DL3*01003i | 1 | 0.00 (0.00,0.01) | 0 | 0.00 (0.00,0.01) | 0 | 0.00 (0.00,0.01) |
| KIR3DL3*01004i | 1 | 0.00 (0.00,0.01) | 1 | 0.00 (0.00,0.02) | 0 | 0.00 (0.00,0.01) |
| KIR3DL3*01101i | 26 | 0.04 (0.03,0.06) | 14 | 0.04 (0.02,0.07) | 12 | 0.05 (0.03,0.08) |
| KIR3DL3*01101i or 012i 02102i | 1 | 0.00 (0.00,0.01) | 1 | 0.00 (0.00,0.02) | 0 | 0.00 (0.00,0.01) |
| KIR3DL3*012i | 8 | 0.01 (0.01,0.03) | 0 | 0.00 (0.00,0.01) | 8 | 0.03 (0.01,0.06) |
| KIR3DL3*012i or 01101i 023i | 2 | 0.00 (0.00,0.01) | 0 | 0.00 (0.00,0.01) | 1 | 0.00 (0.00,0.02) |
| KIR3DL3*01308i | 3 | 0.00 (0.00,0.01) | 2 | 0.01 (0.00,0.02) | 1 | 0.00 (0.00,0.02) |
| KIR3DL3*01408i | 2 | 0.00 (0.00,0.01) | 0 | 0.00 (0.00,0.01) | 2 | 0.01 (0.00,0.03) |
| KIR3DL3*01410i | 1 | 0.00 (0.00,0.01) | 1 | 0.00 (0.00,0.02) | 0 | 0.00 (0.00,0.01) |
| KIR3DL3*01413i | 1 | 0.00 (0.00,0.01) | 1 | 0.00 (0.00,0.02) | 0 | 0.00 (0.00,0.01) |
| KIR3DL3*01502i | 11 | 0.02 (0.01,0.03) | 0 | 0.00 (0.00,0.01) | 9 | 0.04 (0.02,0.07) |
| KIR3DL3*01502i or 012i 01801i | 1 | 0.00 (0.00,0.01) | 0 | 0.00 (0.00,0.01) | 1 | 0.00 (0.00,0.02) |
| KIR3DL3*01502i or 01801i 023i | 1 | 0.00 (0.00,0.01) | 0 | 0.00 (0.00,0.01) | 1 | 0.00 (0.00,0.02) |
| KIR3DL3*01801i | 3 | 0.00 (0.00,0.01) | 2 | 0.01 (0.00,0.02) | 1 | 0.00 (0.00,0.02) |
| KIR3DL3*01801i or 01502i 02102i | 1 | 0.00 (0.00,0.01) | 1 | 0.00 (0.00,0.02) | 0 | 0.00 (0.00,0.01) |
| KIR3DL3*032i | 4 | 0.01 (0.00,0.02) | 1 | 0.00 (0.00,0.02) | 3 | 0.01 (0.00,0.03) |
| KIR3DL3*037i | 1 | 0.00 (0.00,0.01) | 0 | 0.00 (0.00,0.01) | 1 | 0.00 (0.00,0.02) |
| KIR3DL3*040i | 12 | 0.02 (0.01,0.03) | 4 | 0.01 (0.00,0.03) | 8 | 0.03 (0.01,0.06) |
| KIR3DL3*044i | 1 | 0.00 (0.00,0.01) | 1 | 0.00 (0.00,0.02) | 0 | 0.00 (0.00,0.01) |
| KIR3DL3*045i | 3 | 0.00 (0.00,0.01) | 2 | 0.01 (0.00,0.02) | 1 | 0.00 (0.00,0.02) |
| KIR3DL3*052i | 1 | 0.00 (0.00,0.01) | 0 | 0.00 (0.00,0.01) | 1 | 0.00 (0.00,0.02) |
| KIR3DL3*057i or 0040201i 01101i | 3 | 0.00 (0.00,0.01) | 1 | 0.00 (0.00,0.02) | 2 | 0.01 (0.00,0.03) |
| KIR3DL3*058i | 1 | 0.00 (0.00,0.01) | 0 | 0.00 (0.00,0.01) | 1 | 0.00 (0.00,0.02) |
| KIR3DL3*069i or 01101i 0040201i 01101i | 1 | 0.00 (0.00,0.01) | 0 | 0.00 (0.00,0.01) | 1 | 0.00 (0.00,0.02) |
| KIR3DL3*080Ni | 2 | 0.00 (0.00,0.01) | 2 | 0.01 (0.00,0.02) | 0 | 0.00 (0.00,0.01) |
| KIR3DL3*095i | 1 | 0.00 (0.00,0.01) | 1 | 0.00 (0.00,0.02) | 0 | 0.00 (0.00,0.01) |
| KIR3DP1*0030101i | 100 | 0.16 (0.13,0.19) | 48 | 0.14 (0.11,0.19) | 46 | 0.18 (0.14,0.24) |
| KIR3DP1*001i | 382 | 0.61 (0.57,0.65) | 179 | 0.54 (0.48,0.59) | 173 | 0.69 (0.63,0.75) |
| KIR3DP1*00303i | 1 | 0.00 (0.00,0.01) | 1 | 0.00 (0.00,0.02) | 0 | 0.00 (0.00,0.01) |
| KIR3DP1*005i | 109 | 0.17 (0.15,0.21) | 83 | 0.25 (0.20,0.30) | 21 | 0.08 (0.05,0.13) |
| KIR3DP1*00604i | 3 | 0.00 (0.00,0.01) | 2 | 0.01 (0.00,0.02) | 0 | 0.00 (0.00,0.01) |
| KIR3DP1*007i | 2 | 0.00 (0.00,0.01) | 0 | 0.00 (0.00,0.01) | 2 | 0.01 (0.00,0.03) |
| KIR3DP1*008i | 1 | 0.00 (0.00,0.01) | 0 | 0.00 (0.00,0.01) | 1 | 0.00 (0.00,0.02) |
| KIR3DP1*00902i | 1 | 0.00 (0.00,0.01) | 0 | 0.00 (0.00,0.01) | 1 | 0.00 (0.00,0.02) |
| KIR3DP1*0140101i | 19 | 0.03 (0.02,0.05) | 16 | 0.05 (0.03,0.08) | 3 | 0.01 (0.00,0.03) |
| KIR3DP1*017i | 3 | 0.00 (0.00,0.01) | 0 | 0.00 (0.00,0.01) | 3 | 0.01 (0.00,0.03) |
| KIR3DP1*023i | 1 | 0.00 (0.00,0.01) | 1 | 0.00 (0.00,0.02) | 0 | 0.00 (0.00,0.01) |
| KIR3DP1*029i | 2 | 0.00 (0.00,0.01) | 0 | 0.00 (0.00,0.01) | 0 | 0.00 (0.00,0.01) |
| KIR3DS1*010i | 182 | 0.29 (0.26,0.33) | 126 | 0.38 (0.33,0.43) | 34 | 0.14 (0.10,0.18) |
| KIR3DS1*049Ni | 12 | 0.02 (0.01,0.03) | 8 | 0.02 (0.01,0.05) | 4 | 0.02 (0.00,0.04) |

**Supplement Table 2**:

Determination of race and ancestry were assessed by patient self-report and genetic variation using the Axiom Precision Medicine Diversity Research Array(PMDRA).^46^ Due to DNA availability, only 398 of 506 subjects could be genotype using the PMDRA. PMRDA interrogates biallelic single nucleotide polymorphisms (SNPs) and simple indels. A principal components analysis (PCA) approach, using the first four principal components (PC), was used to model population stratification. These analyses were conducted using R version 4.1.3 base and libraries rsvd, plyr, haven, and ggplot2. For example, using logistic regression, the first 4 PCs predicted White race as compared to non-White with a receiver operator curve area of 0.96. As shown in the table below, our results were minimally changed when 4 PCAs were included in the regression models. The ORs change very little after the PCA correction. The 95% CI and p-values do change as expected as the sample size is smaller (398 instead of 506) and four additional covariates were included in the model which can reduce efficiency.

Importantly, this is a supportive analysis and not our primary analysis. However, our results are consistent with, as noted in the methods section, previous knowledge of genetic admixture and its influence on gene associations in AD.

**Supplement Table 2**: Odds ratios and 95% CI for the association of atopic dermatitis by KIR alleles of interest. Associations are presented as compared to no gene and as no allele (gene present but not the specific allele). The first column is as reported in Table 2 of the manuscript and the second column includes an adjustment for the first 4 principal components *p < 0.05, ^p <0.005, ~p <0.001

|  | Per Table 2  OR (95% CI) | PCA adjusted  OR (95% CI) |
| --- | --- | --- |
| KIR2DL5A | 1.17 (0.72,1.90) | 0.96(0.54,1.70) |
| KIR2DL5B | 2.09 (1.28,3.41)^ | 2.18(1.18,4.02)* |
| KIR2DL5*002:01:01 | 2.18 (1.34,3.53)~ | 2.16(1.18,3.94)* |
| KIR2DL5*001:01:01 | 1.22 (0.75,1.98) | 0.96 (0.54,1.70) |
| KIR2DS1*002:01:01 | n/a | n/a |
| KIR2DS5*002:01:01 | 2.91 (1.24,6.80)* | 2.43(0.76,7.79) |
| KIR2DS5*005:01 | 1.41 (0.93,2.14) | 1.65(1.00,2.74) |
